# How identity bias affects perceptions of conservation messages on social media

**DOI:** 10.1111/cobi.70315

**Published:** 2026-05-08

**Authors:** Lauren F. Rudd, Yolanda Mutinhima, Shorna Allred, Amy J. Dickman, Darragh Hare

**Affiliations:** ^1^ Department of Biology University of Oxford Oxford UK; ^2^ Department of Wildlife Ecology and Conservation Chinhoyi University of Technology Chinhoyi Zimbabwe; ^3^ Centre for Sustainability Transitions Stellenbosch University Stellenbosch South Africa; ^4^ Department of Geography and Environment University of North Carolina at Chapel Hill Chapel Hill North Carolina USA; ^5^ Wildlife Conservation Research Unit, Department of Biology University of Oxford Oxford UK; ^6^ Lion Landscapes Teignmouth UK; ^7^ Department of Natural Resources and the Environment Cornell University Ithaca New York USA

**Keywords:** bias, conservation messaging, discrimination, identity, public, racism, sexism, social media, discriminación, identidad, mensajes de conservación, opinión pública, redes sociales, sesgos, sexismo, 偏见, 身份, 歧视, 社交媒体, 公众, 种族主义, 性别歧视, 保护信息传播

## Abstract

Public support is essential for conservation, as public opinion can influence decision‐making and policy. Therefore, understanding whether bias toward conservationists due to their identity (identity bias) affects their perceived credibility and support for their recommendations is important. We conducted a vignette‐style experiment to investigate the extent to which identity bias influences the U.K. public's perceptions of African lion (*Panthera leo*) conservation messengers on X. We chose this focal species because of its global appeal and high public engagement in lion conservation on social media. We created 24 fictitious X profiles with a pinned post about lion conservation and presented one profile to each of 1290 study participants (stratified to match the U.K. population regarding age, gender identity, and ethnicity). We held the lion conservation post constant across all profiles but manipulated four experimental variables—the gender, race, expertise, and nationality of the messenger. We evaluated the effects of these variables on three response variables: respondents’ perception of the credibility of post content, the likelihood they would support implementing the lion conservation recommendation, and the trustworthiness of the messenger. Men were perceived to be more credible than women. Support for implementing a lion conservation strategy was stronger when communicated by White professors than by Black professors. Explicit trust in the source of lion conservation information followed the same pattern. Additionally, there was an interaction effect of gender and race, with Black women perceived to be the least trustworthy. As such, our study highlights that bias against conservation messengers based on their identity may affect their credibility and uptake of their recommendations. Such bias is particularly concerning given the ongoing injustices and entrenched power inequalities in global conservation efforts.

## Abstract (es)

Cómo influye el sesgo de identidad en la percepción de los mensajes sobre conservación en las redes sociales

**Resumen:**

El apoyo público es esencial para la conservación, ya que la opinión pública puede influir en la toma de decisiones y en las políticas. Por lo tanto, es importante comprender si el sesgo hacia los conservacionistas debido a su identidad (sesgo de identidad) afecta a la credibilidad que se les atribuye y al apoyo que reciben sus recomendaciones. Llevamos a cabo un experimento basado en viñetas para investigar en qué medida el sesgo de identidad influye en la percepción que tiene el público del Reino Unido de los defensores de la conservación del león africano (*Panthera leo*) en X. Elegimos esta especie como objeto de estudio debido a su atractivo global y al alto nivel de participación pública en la conservación del león en las redes sociales. Creamos 24 perfiles ficticios en X con una publicación destacada sobre la conservación del león y presentamos un perfil a cada uno de los 1290 participantes en el estudio (estratificados para reflejar la población del Reino Unido en cuanto a edad, identidad de género y origen étnico). Mantuvimos constante la publicación sobre la conservación de los leones en todos los perfiles, pero manipulamos cuatro variables experimentales: el género, la raza, la experiencia y la nacionalidad del emisor. Evaluamos los efectos de estas variables en tres variables de respuesta: la percepción de los encuestados sobre la credibilidad del contenido de la publicación, la probabilidad de que apoyaran la implementación de la recomendación de conservación de los leones y la fiabilidad del emisor. Se percibió a los hombres como más crédulos que a las mujeres. El apoyo a la implementación de una estrategia de conservación de los leones fue mayor cuando la comunicaban profesores blancos que cuando lo hacían profesores negros. La confianza explícita en la fuente de la información sobre la conservación de los leones siguió el mismo patrón. Además, se observó un efecto de interacción entre género y raza, y se percibió a las mujeres negras como las menos fiables. Por lo tanto, nuestro estudio pone de relieve que los prejuicios contra los mensajeros de la conservación basados en su identidad pueden afectar a su credibilidad y a la aceptación de sus recomendaciones. Dichos prejuicios son especialmente preocupantes dadas las injusticias actuales y las arraigadas desigualdades de poder en los esfuerzos de conservación a nivel mundial.

## 身份偏见如何影响社交媒体上公众对保护信息的认知

**【摘要】**公众支持对保护工作至关重要, 因为公众舆论会影响决策和政策制定。因此, 了解公众对保护主义者身份的偏见(身份偏见)是否会影响对其可信度的认知以及对其建议的支持程度, 具有重要意义。本研究开展了一项情景实验, 旨在探究身份偏见在多大程度上影响英国公众对X平台上非洲狮(*Panthera leo*)保护传播者的认知。我们选择该焦点物种是因为其具有全球吸引力, 且社交媒体上公众对狮子保护的参与度很高。我们创建了24个虚构的X平台账号, 每个账号均置顶了一条关于狮子保护的帖子, 并将其中一个账号展示给1290名研究参与者(根据英国人口结构对年龄、性别认同和种族进行分层抽样)。所有账号的狮子保护帖子内容均相同, 但我们操纵了四个实验变量, 分别是传播者的性别、种族、专业知识和国籍。我们评估了这些变量对三个响应变量的影响, 包括受访者对帖子内容可信度的认识、受访者支持实施狮子保护建议的可能性, 以及信息发布者的可信度。结果显示, 男性被认为比女性更可信。相比于黑人教授, 当信息由白人教授发布时, 人们对实施狮子保护策略的支持度更高。受访者对狮子保护信息来源的明确信任度也呈现出相同的模式。此外, 性别与种族之间存在交互作用, 其中黑人女性被认为最不可信。因此, 本研究强调, 对保护信息传播者的身份偏见可能影响其可信度及其建议的被采纳情况。鉴于全球保护工作中持续存在的不公正现象和根深蒂固的权力不平等, 此类偏见尤为令人担忧。**【翻译:胡怡思;审校:聂永刚】**

## INTRODUCTION

Many species are under threat of extinction, and effective conservation requires well‐informed decision‐making (Ceballos et al., 2017; Nielsen et al., 2021). However, conservation decisions and policy are affected by the views of multiple publics, who may form their judgments based on different factors (Duthie et al., 2017; Massingham et al., 2023; Wright et al., 2015). Public buy‐in to conservation is vital (Crowley et al., 2017) but can be contentious and challenging when the species of concern are highly valued and under substantial threat (Macdonald et al., 2015).

One such example is the African lion (*Panthera leo*), classified as vulnerable on the IUCN Red List and predominantly threatened by habitat fragmentation, conflict with people, and prey base depletion (Bauer et al., 2016). Strategies for conserving lion populations must balance the ecological needs of the species alongside the socioeconomic dynamics of the landscapes within their range (Bauer et al., 2022; Lindsey et al., 2018). In particular, the success of future conservation efforts hinges on properly acknowledging and addressing colonial histories of Eurocentric conservation across lion ranges (Kimaro & Hughes, 2025). Globally, the African lion holds immense cultural significance due to its high existence value (Macdonald et al., 2015), meaning that public engagement in the discourse around lion conservation is high even outside its present range (Macdonald et al., 2016; McCubbin, 2020). Given the complex and often controversial nature of lion conservation strategies, heated debates between stakeholders, scientists, policy makers, and members of multiple publics—all with competing visions for how to best conserve lions—are frequent. Although disagreements between scientists and stakeholders are often documented in academic publications (Chimuka, 2022; Hart et al., 2020; Macdonald et al., 2016; Nelson et al., 2016; Van Eeden et al., 2017), knowledge of debates within the public sphere (of any geographic location) is less well understood.

Much public discourse about lion conservation takes place on social media platforms, such as X (formerly Twitter) (Chimuka, 2022; Evans et al., 2023; Macdonald et al., 2016), where the credibility of topical scientific information can be hard to establish (Yang et al., 2021) and the spread of misinformation is prolific (Vosoughi et al., 2018). As a consequence, debates can lack nuance and descend into verbal abuse directed toward those perceived to be harming wildlife (Lunstrum, 2017) or become focused on division rather than finding mutually beneficial ways forward (Mkono, 2022). Often, such online public attention is dominated by distant publics in high‐income countries directed toward conservation action in lower‐income countries, with little consideration of local perspectives, such as in recent conversations around trophy hunting (McCubbin, 2020).

As conservation can depend on public donations and support, public opinion may influence policy‐relevant decision‐making and outcomes (Hare et al., 2024; Lunstrum, 2017; Martín‐López et al., 2009). Such public opinion can be influenced by a suite of factors, including individuals’ values and attitudes (Frater et al., 2025). Message framing may also influence people's perceptions of conservation issues (Kidd et al., 2019), as have perceptions of the messenger themselves (Mathiesen et al., 2022; Sauer et al., 2021). For example, research has shown that the perception of veterinarians as trusted sources of advice plays a key role in encouraging cat owners to bring their cats inside overnight to keep wildlife safe (Elliott et al., 2019). It is therefore critically important to understand whether and how, when discussing conservation recommendations on platforms such as X, bias toward individuals due to their identity (e.g., their race, gender) (referred to from here on as *identity bias*) affects their perceived credibility.

Evidence of identity bias affecting behavior on social media has been observed in fields outside conservation, where the gender and race of broadcasters affect their perceived objectivity by the public (Boling & Walker, 2021). Similarly, the race and physical attractiveness of an individual's profile photo have been found to affect public evaluations of trust in them, as well as the likelihood of following them (Groggel et al., 2019). Any such bias against conservationists might affect decision‐making and the direction of conservation resources. Ultimately, this could influence outcomes for people, their livelihoods, and wildlife (Evans et al., 2023; Hammond et al., 2022). Such effects could be particularly damaging if compounded by conservation's entrenched power inequalities, such as systematic prejudice against local communities and Indigenous peoples (Domínguez & Luoma, 2020; Kashwan et al., 2021).

In addition, race and gender inequity within the professional conservation sphere (James et al., 2023; Rudd et al., 2021; Taylor, 2015) create a lack of visible diversity. Questions have been raised regarding the value placed on academic or scientific qualifications compared to practical experience and contextual knowledge of conservation problems (Ashley‐Smith, 2016; Fernández‐Llamazares et al., 2021; Malmer et al., 2020; Orlove et al., 2023). This can often be compounded by parachute science (Haelewaters et al., 2021), when researchers from wealthy countries exploit the expertise and resources from comparatively poor countries and do not properly involve local collaborators. Taken together, these factors may create a stereotypical image in the public eye of what a conservation expert looks like (Miele, 2014). In turn, this could influence whose opinions are perceived as credible, and therefore trusted and supported, when debating contentious conservation issues online.

We report results from an online questionnaire experimentally investigating the effects of identity bias toward conservation messengers on X, among X users living in the United Kingdom (at the time of the study, the platform was branded as Twitter). Specifically, we examined whether the race, nationality, gender, and level of expertise of those posting on X about lion conservation could influence perceived credibility of the lion conservation strategy they endorsed, support for implementing that strategy, and perceived trustworthiness of the source regarding lion conservation information. We evaluated whether and how identity bias affects explicit perceptions of those sharing conservation messages, and the variation this generates in public engagement with such messaging. By identifying and quantifying such biases, we provide evidence to help rectify insidious problems of bias and inequity in global conservation efforts.

## METHODS

### Questionnaire development and sampling

To test our survey instruments, we consulted a group of academics (*n* = 8) with expertise in conservation, human behavior, environmental justice, equality, and diversity and inclusion. We incorporated their comments and suggestions into the questionnaire before data collection began. The final version of the questionnaire is in Appendix S1.

Using Qualtrics XM, we recruited 1290 participants aged 18 and over living in the United Kingdom from 6 September to 8 November 2022 to respond to our survey, also hosted on the Qualtrics platform. To ensure our results were indicative of the adult U.K. population, we stratified our sample to match the most recently published statistics (at the time) on age, ethnicity, and gender identity (Office for National Statistics, 2021) (Appendix S2). All respondents were financially compensated for their time.

### Experimental design

**FIGURE 1:**
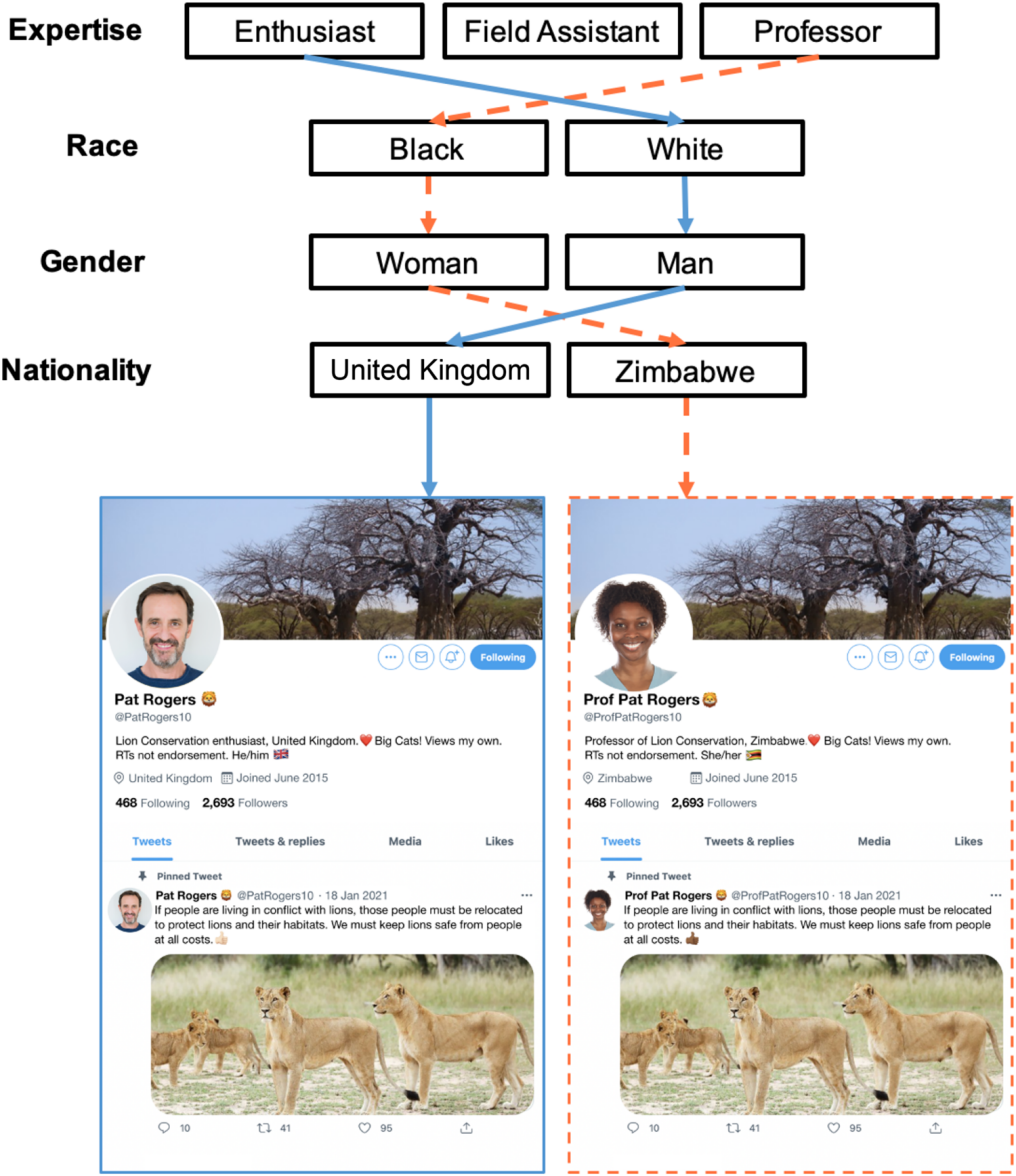
The experimental design of a study investigating the extent to which identity bias influences the U.K. public's perceptions of African lion (*Panthera leo*) conservation messengers on X. Profiles shown are example X profiles. Pictures (X profile and pinned post) shown to respondents were identical apart from the specifics related to each factor that was manipulated: expertise, race, gender, nationality, and location tag.

We created snapshots of fictitious Twitter profiles (now referred to as X profiles), from cover photo to a pinned tweet (now referred to as a pinned post) with Inkscape. We manipulated the profile photo (sourced on Shutterstock), emojis, and the language within the character's biography to indicate their gender (man or woman), race (Black or White), nationality or location (Zimbabwe or the United Kingdom), and expertise (professor, field assistant, or enthusiast), chosen to represent varied levels of knowledge, both formal and informal (Figure 1). Images of all profiles created are in Appendix S3. A full factorial design (2 × 2 × 2 × 3) resulted in 24 experimental conditions, each represented by a unique character embedded within the otherwise uniform image of an X profile. We took care when selecting the four different profile photos to ensure characters appeared to be of a similar age and the photos were presenting the person in a similar way (directly facing the camera, smiling, visible from the shoulders up, and against a plain background). We used the name Pat Rogers for all characters.

Although we expected the content of the lion conservation message to affect public perceptions of credibility, trustworthiness, and support, evaluating the effect of different messages was not our research objective. Thus, we held the message constant. To determine which lion conservation message to use as the pinned post (to allow us to best interpret any effect of identity bias), we first conducted a preliminary message‐selection study (Appendix S3). Based on these results, the pinned post read “If people are living in conflict with lions, those people must be relocated to protect lions and their habitats. We must keep lions safe from people at all costs.” and was held constant across all treatments. The questions were presented to all respondents in the same order, and they were unable to return to a previous question to change their answer. This allowed us to ensure that the effects of priming were uniform across all vignettes (Strack, 1992).

### Procedure

Respondents first answered a block of sociodemographic questions to ensure they met the study quotas set for age, ethnicity, and gender identity, as well as a screening question about their X usage in the past 12 months. Any who did not fit the quotas or who had not used X in the previous 12 months were not invited to continue. Next, respondents were presented with a short informative passage about lion conservation and management strategies (Appendix S1). This was designed to be objective, provide context, and present an unbiased overview that ensured all respondents had sufficient information to answer the questionnaire. It was written in consultation with lion conservation researchers and based on published scientific information.

Each respondent was randomly assigned to one of 24 possible experimental conditions. The image of the X profile they were assigned remained visible throughout the portions of the questionnaire that it related to. Respondents answered three questions used to measure credibility, support, and trustworthiness. Answers were on a 7‐point Likert‐type scale ranging from *strongly disagree* to *strongly agree*; the midpoint was “neither agree nor disagree.” The response “I don't know” was also an option.

To measure perceived credibility, support for the recommendation, and perceived trustworthiness, we asked respondents to what extent do you agree that the information presented in Pat Rogers’ pinned post is credible (measured credibility) (Question 1); to what extent do you agree with Pat Rogers that we should relocate people to address human–lion conflict (measured support) (Question 2); and to what extent do you agree that Pat Rogers is a trustworthy source of lion conservation information (measured trustworthiness) (Question 3).

Participants then provided information on their level of X usage, formal education, the extent to which they supported human rights and animal rights, how they would prioritize the interests of people versus the interests of wild animals when their interests clashed (Hare et al., 2024), and how much confidence they had in scientists to act in the best interests of the public. The full questionnaire is available in Appendix S1.

The study received ethics clearance by a subcommittee of the University of Oxford Central University Research Ethics Committee (R79948/RE001), and all respondents provided informed consent prior to answering any questions.

### Data analyses

We analyzed data with R 4.2.0. A detailed breakdown describing the sample, including demographic characteristics and covariates, can be found in Appendix S2. The median response time was 176.5 s, and we removed all responses from participants who took less than half or more than four times the median prior to analysis. The total number of responses was 1290. Respondents who answered “I don't know” to one or more of the three questionnaire items measuring credibility, support, and trustworthiness were removed prior to conducting the analysis for that question. The resultant sample sizes were 1254 responses for Question 1, 1260 responses for Question 2, and 1178 for Question 3. We used the likert package (Bryer & Speerschneider, 2016) to visualize raw data.

Using the ordinal package (Christensen, 2023), we fitted separate ordinal logistic regression models to quantify relationships between the experimental factors manipulated within the vignettes (gender, race, nationality, and expertise) and credibility, support, and trustworthiness. For each analysis, we first fitted a global model containing all experimental factors and all possible interactions between these factors, as well as respondents’ age, gender identity, ethnicity, extent of X usage, how much confidence they have in scientists to act in the public's interest, the extent to which they support human rights and animal rights, and how they would prioritize the interests of wild animals versus people. Owing to the low number of participants identifying with certain ethnicities, all responses were condensed into broader ethnicity categories of White; Asian or Asian British; Black, African, Caribbean, or Black British; mixed or multiple ethnic groups; other ethnic groups; and prefer not to say. These categories are consistent with the classification system used by the Office for National Statistics in the United Kingdom (Office for National Statistics, 2021) (Appendix S2).

Using the MuMIn package (Barton, 2023), we compared corrected Akaike information criterion (AIC_c_) values for each global model and all possible nested models within it. The top‐supported model for each response variable was identified as that with the lowest AIC_c_. After removing uninformative and redundant parameters (Arnold, 2010; Leroux, 2019), we calculated AIC_c_ weights for all top‐supported models (those within Δ2 AIC_c_). We reported parameter estimates, 85% confidence intervals (CIs), and 85% and 95% CIs in all figures following best practice for model selection based on information theory (Sutherland et al., 2023). We conducted post hoc Tukey tests with the emmeans package (Lenth, 2024) to evaluate the effects of each level of our predictor variables within the interaction terms. We used ggplot2 (Wickham, 2024) to visualize these interactions and the coefficient estimates for the predictors in each top‐supported model.

## RESULTS

### Credibility of the pinned post

**FIGURE 2:**
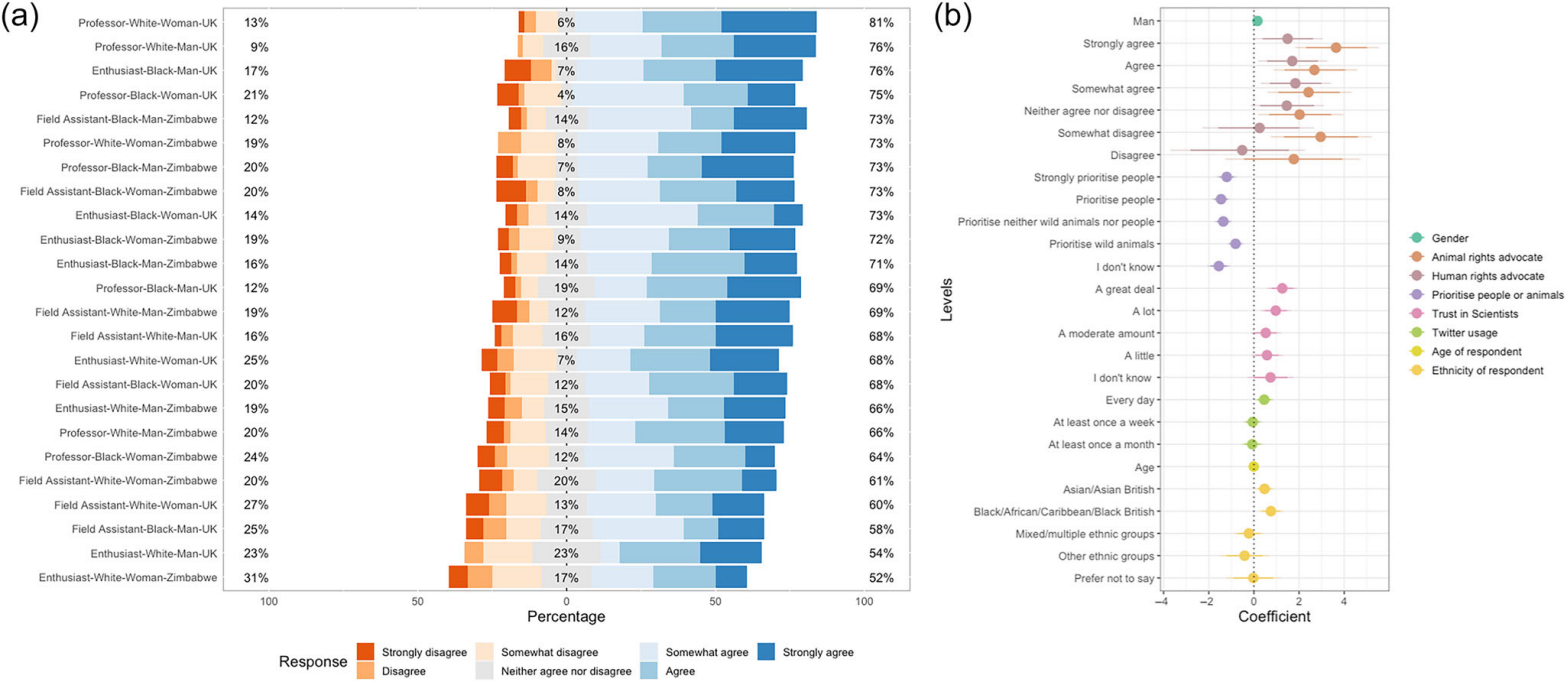
(a) Extent of agreement of survey respondents (n = 1254 respondents aged 18 and over living in the United Kingdom, stratified to match the population based on age, ethnicity, and gender identity) that the information in fictitious expert Pat Rogers’ pinned tweet is credible based on Rogers’ experimental profile, in which level of expertise, race, gender, and nationality are indicated (bars, responses to one of the 24 profiles tested; percentages on the left, percentage of respondents who overall disagreed; middle, were neutral; right, agreed; “I don't know” responses excluded) and (b) associations between agreement that Pat Rogers pinned tweet is credible and the variables (at right) in the top‐supported model (points, coefficient estimates for the levels of variables relative to the reference category for categorical variables [reference state: gender, woman; animal and human rights advocates, strongly disagree; prioritize people or animals, strongly prioritize wild animals; trust in scientists, no confidence; Twitter usage, less than once a month; ethnicity, English, Welsh, Scottish, Northern Irish, or British]; positive coefficients, higher level of agreement than reference level; negative coefficients, lower level of agreement than reference level; whiskers, 85% CIs; transparent extensions, 95% CIs).

Agreement with the credibility of Pat Rogers’ pinned post varied depending on the character presented (Figure 2a). Our top‐supported model of credibility (AIC_c_ weight = 0.22; Appendix S4.1) contained a main effect of one experimental factor, the gender of Pat Rogers. Holding all other predictors constant, respondents were more likely to agree that the pinned post was credible if it was embedded within the profile of a man rather than a woman (log odds ratio [SE] = 0.157 [0.103]) (Figure 2b) (all log odds ratios are in Appendix S5).

The model also contained effects of the respondent's support for animal rights, support for human rights, beliefs about whether to prioritize people or animals, the extent of their trust in scientists, level of X usage, their age, and ethnicity (Appendix S5). Respondents who strongly supported animal rights were more likely to agree that the pinned post was credible (log odds ratio [SE] = 3.636 [0.925]). Conversely, respondents were less likely to agree that the pinned post was credible if they strongly prioritized people over animals (log odds ratio = −1.207 [0.224]). In addition, respondents with greater trust in scientists to act in the public interest were more likely to agree that the pinned post was credible compared with those who had no trust at all (log odds ratio = 1.255 [0.338]). Those who used X daily were also more likely to agree that the pinned post was credible compared with those who used it less than once a month (log odds ratio = 0.454 [0.212]). Older respondents were less likely to agree that the pinned post was credible (log odds ratio = −0.01 [0.003]) (Figure 2b; Appendix S5).

Despite being in the model, the relationship between respondents’ agreement with the credibility of the pinned post and their support for human rights was not straightforward to interpret. This was also true of the relationship with respondents’ ethnicity. In both cases, this was likely due to small numbers of respondents in some of the categories included (Figure 2b; Appendices S2 & S5).

### Support for the position taken (relocating people)

**FIGURE 3:**
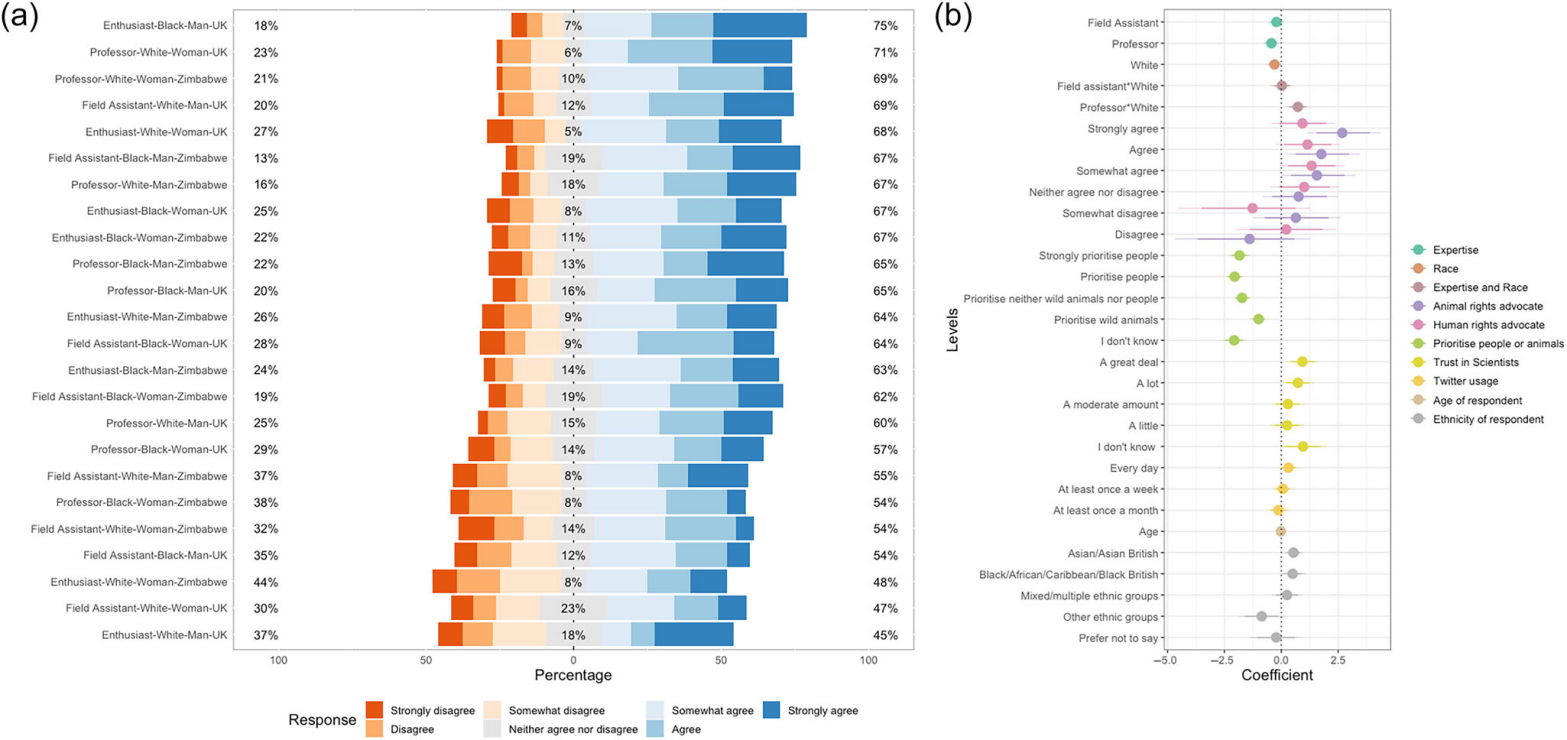
(a) Extent of agreement of survey respondents (n = 1260 respondents aged 18 and over living in the United Kingdom, stratified to match the population based on age, ethnicity, and gender identity) with fictitious expert, Pat Rogers, that people should be relocated to protect lions based on the Rogers’ experimental profile (level of expertise, race, gender, and nationality) (bars, responses to one of the 24 profiles being tested; percentages on the left, percentage of respondents who overall disagreed; middle, were neutral; right, agreed; “I don't know” responses excluded) and (b) associations between agreement with Pat Rogers that lions should be relocated to protect people and the variables (at right) in the top‐supported model (points, coefficient estimates for the levels of each variable relative to the reference state [expertise, enthusiast; race, Black; animal rights advocate and human rights advocate, strongly disagree; prioritize people or animals, strongly prioritize wild animals; trust in scientists, no confidence; Twitter usage, less than once a month; ethnicity, English, Welsh, Scottish, Northern Irish, British]; positive coefficients, higher level of agreement than reference level; negative coefficients, lower level of agreement than reference level; whiskers, 85% CIs; transparent extensions, 95% CIs).

Support for the character's stated position (in this case, relocating people to address human–lion conflict) varied depending on the character sharing the message (Figure 3a). The top‐supported model (AIC_c_ weight = 0.35) (Appendix S4.2) contained an interaction between two experimental factors, the race and expertise of Pat Rogers (Figure 3b, with all log odds ratios and SE available in Appendix S6.1), meaning the influence of expertise depended on race.

**FIGURE 4:**
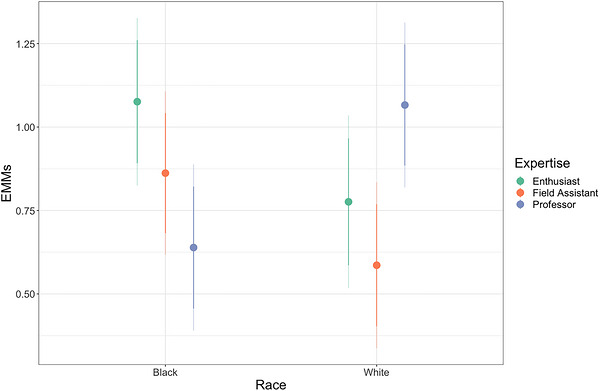
Estimated marginal means (EMMs) for the interaction between race and level of expertise in the top‐supported model for the question to what extent do you agree with Pat Rogers that people should be relocated to protect lions (Question 2) based on n = 1260 survey respondents aged 18 and over living in the United Kingdom (stratified to match the population based on age, ethnicity, and gender identity) (the higher the EMM, the more positive the effect of that combination of factor levels on the level of agreement; whiskers, 85% CI; transparent extensions, 95% CIs).

When Pat Rogers was Black, respondents were most likely to support the relocation of people if they were an enthusiast (Tukey test, estimate [SE] = 1.076 [0.127]), followed by a field assistant (estimate = 0.862 [0.125]), and were least likely to support a professor (estimate = 0.639 [0.127]) (Figure 4; Appendix S6.2). In contrast, when Pat Rogers was White, the order was reversed: support for relocating people was highest when Pat was a professor (estimate = 1.066 [0.126]), followed by an enthusiast (estimate = 0.776 [0.132]), and lowest for a field assistant (estimate = 0.586 [0.127]) (Figure 4; Appendix S6.2).

The top‐supported model contained effects of respondents’ support for animal rights and human rights, their beliefs about the prioritization of people versus animals, the extent of their trust in scientists, their level of X usage, their age, and their ethnicity. These factors were all similarly contained in the top‐supported model for the perceived credibility of the pinned post, and the relationships between the levels of these factors and support for relocating people were nearly identical to those outlined in the “Credibility of the pinned post” section (Appendix S6.1).

### Trustworthiness of Pat Rogers

**FIGURE 5:**
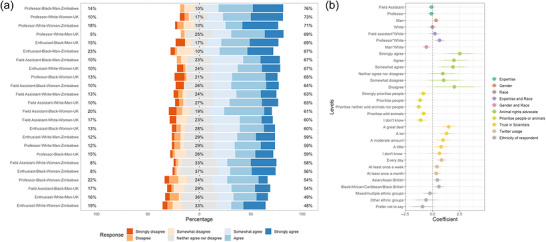
(a) Agreement of survey respondents (n = 1178 respondents aged 18 and over living in the United Kingdom, stratified to match the population based on age, ethnicity, and gender identity) that a fictitious expert, Pat Rogers, is a trustworthy source of lion conservation information based on experimental profile of Rogers (level of expertise, race, gender, nationality) (bars, distribution of responses to one of the 24 profiles tested; percentages on the left, percentage of respondents who overall disagreed; middle, were neutral; right, agreed; “I don't know” responses excluded) and (b) associations between agreement that Pat Rogers is a trustworthy source of lion conservation information and variables (at right) in the top‐supported model (points, coefficient estimates relative to the reference state [expertise, enthusiast; gender, woman; race, Black; expertise and race, enthusiast White; gender and race, woman White; animal rights advocate, strongly disagree; prioritize people or animals; strongly prioritize wild animals; trust in scientists, no confidence; Twitter usage, less than once a month; ethnicity, English, Welsh, Scottish, Northern Irish, British]; positive coefficients, level of agreement higher than reference level; negative coefficients, level of agreement lower than reference level; whiskers, 85% CIs; transparent extensions, 95% CIs).

Agreement that Pat Rogers was a trustworthy source of lion conservation information varied depending on the character presented (Figure 5a). The top‐supported model (AIC_c_ weight = 0.23) (Appendix S4.3) contained the two‐way interactions between the race and expertise, and the race and gender of Pat Rogers (Figure 5b) (all log odds ratios are in Appendix S7.1), meaning the extent of trust in Pat Rogers’ expertise was dependent on their race.

**FIGURE 6:**
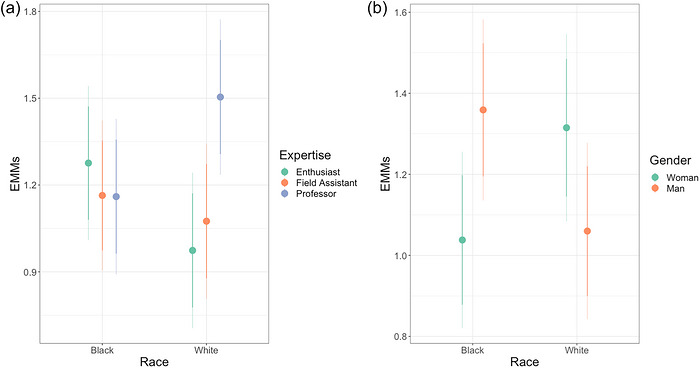
(a) Associations between race and levels of expertise and (b) association between race and gender (points, estimated marginal means [EMMs] for the interactions in the top‐supported model for the question to what extent do you agree that Pat Rogers is a trustworthy source of lion conservation information [Question 3] based on n = 1178 survey respondents aged 18 and over living in the United Kingdom [stratified to match the population based on age, ethnicity, and gender identity]) (the higher the EMM, the more positive effect of that combination of factor levels on increasing levels of agreement; whiskers, 85% CIs; transparent extensions, 95% CIs).

Professor was the most trusted expertise level when Pat Rogers was White (Tukey test, estimate [SE] = 1.504 [0.137]) but the least trusted when they were Black (estimate = 1.160 [0.137]) (Figure 6a; Appendix S7.2). Conversely, the enthusiast was perceived as the most trustworthy when Pat Rogers was Black (estimates = 1.276 [0.136]) but the least trustworthy when they were White (estimates = 0.974 [0.137]) (Figure 6a; Appendix S7.2). Trust of the Black field assistant and White field assistant sat in the middle (estimates = 1.164 [0.132] and 1.075 [0.137], respectively) (Figure 6a; Appendix S7.2).

The influence of gender on respondents’ trust in Pat Rogers was further dependent on Pat Rogers’ race. When Pat Rogers was a man, they were perceived as more trustworthy when they were Black (Tukey test, estimate [SE] = 1.359 [0.114]) than when they were White (estimate = 1.060 [0.111]) (Figure 6b; Appendix S7.2). However, the opposite was true when Pat Rogers was a woman, with respondents perceiving the White character as more trustworthy than the Black character (estimates = 1.315 [0.118] and 1.038 [0.111], respectively) (Figure 6b; Appendix S7.2).

The demographic and social identity factors in the top‐supported model included extent of support for animal rights, their beliefs about the prioritization of people versus animals, their extent of trust in scientists, level of X usage, and ethnicity. The relationships between the levels of these factors and agreement that Pat Rogers was a trustworthy source of lion conservation information were nearly identical to those in the previous models (Appendix S7.1).

## DISCUSSION

Across a sample stratified to approximate the U.K. public, we found evidence that identity bias influenced the perceived credibility of lion conservation information, support for acting on that information, and the trustworthiness of a conservation messenger. Specifically, we found bias relating to the race, gender, and expertise of the conservation messenger but not their nationality. This could be due to the lack of immediate visibility of the character's nationality in the profiles created, as it was indicated via a small flag emoji in the profile biography and via the location tag.

Overall, men were perceived to have posted more credible lion conservation information than women. However, gender did not explain support for implementing the conservation strategy in the pinned post. Gender bias, its impact within the field of conservation, and recommendations for addressing such inequities have been well documented in the literature (Giakoumi et al., 2021; James et al., 2021, 2023; Lau, 2020; Liévano‐Latorre et al., 2020). On X, women in other scientific fields have fewer followers, reposts, and likes compared with men (Zhu et al., 2019). This relationship is also found in the Altmetric scores of papers, with higher scores attributed to those published with lead authors who are men versus women (Chapman et al., 2022). We considered the nuances of such gender bias in conservation discourse on X. Although the public perceives women as less credible when discussing conservation, this bias did not significantly affect public support for their recommendations.

Men use stronger language to promote the importance of their scientific research than do women (Lerchenmueller et al., 2019). By holding the messaging constant across all treatments in our study, the results demonstrated that bias against women speaking about conservation exists irrespective of the way in which they convey their opinion. Our results indicate a need to consider the potential for additional impacts of gender bias on public perceptions of messengers on social media, such as funding distribution and public engagement opportunities (McKinnon & O'Connell, 2020), including how these might interact with race and the conservation context presented. We used only binary gender, but conducting similar studies with a greater spectrum of gender identities would further enrich understanding. In addition, we did not examine the presence or absence of same‐gender affinity effects between the respondent and the messenger. There exists evidence that social messaging is more effective from same‐gender influencers (Hudders & De Jans, 2022), presenting a potentially fruitful avenue for future research in conservation messaging.

White women were perceived as more explicitly trusted sources of lion conservation information than White men, even though men were perceived to have posted more credible information. The reverse was true when Pat Rogers was Black, with men being perceived as more trustworthy sources of lion conservation information than women (see Figure 4b for more detail). This highlights the importance of using an intersectional lens when examining patterns of bias in conservation (Crenshaw, 1997; Rosette et al., 2018). The devaluing of Black women's opinions and expertise, combined with inflated workloads comparable to those their White colleagues in academia, has been well documented (Bumpus, 2020; Gewin, 2020; Wright et al., 2007). It is possible that chronic devaluation, combined with racial stereotyping (related to inferred nationality and presumptions of local knowledge, which is discussed later in this section), is driving the observed gender–race interaction patterns.

We found no effect of expertise, indicated by a professional title, on the perceived credibility of conservation information shared via a post on X. Although surprising, it suggests that rejections of conventional scientific expertise are prolific on X, which aligns with the well‐documented, abundant, and rapid spread of science misinformation on social media platforms (Vosoughi et al., 2018). It could also be indicative of an increasing public acknowledgment and recognition of multiple ways of knowing outside of formally qualified experts (Fernández‐Llamazares et al., 2021; Malmer et al., 2020; Orlove et al., 2023). However, we did find an effect of the interaction between the expertise and race of the messenger on both support for relocating people to protect lions and the character's perceived trustworthiness. The White professor received considerably more support and trust than the Black professor. Furthermore, the Black professor was both the least supported and trusted when compared with the Black field assistant and enthusiast, whereas the White professor was consistently the most supported and trusted compared with the White field assistant and enthusiast.

These findings likely stem from societal racism and entrenched stereotypes about who is seen as a conservation expert among the U.K. public (Miele, 2014). This is particularly likely given the low Black representation among senior academics, and so professors, in U.K. higher education institutions (UK Research & Innovation, 2023). Our results highlight potential real‐world implications of this, with the societal prestige of a White professor not afforded equally to those who are Black. Additionally, our results may reflect public exposure to biased conservation narratives in popular media, which often center White individuals as the scientific experts (Abidin et al., 2020; Brockington, 2009; McCubbin, 2020).

It is possible that the greater support for and trust in Pat Rogers as an enthusiast and field assistant when they were Black, compared with when they were White, was due to assumptions about nationality due to race, and thus inferred local contextual understanding of lion conservation. We varied nationality across both the Black and White character X profiles and did not detect an effect, but this could have been due to racial stereotypes superseding the way we signaled nationality. To investigate, further research could explicitly test intersectional identity dynamics by signaling nationality more clearly. It would further be interesting to examine how support varies based on the inferred location of the conservation issue being discussed and the race of the person discussing it. It would be valuable to examine any impacts of racial affinity bias (in favor of individuals one perceives as being like oneself) between each respondent and the messenger based on shared identity. This would require a larger sample size of respondents from minority ethnicities, rather than a respondent pool stratified to match the ethnic demographics of the adult U.K. population.

Combined, our findings indicate the potential for extensive ramifications given the ability of public opinion to sway conservation policy and funding decisions (Anderson et al., 2017; Larson et al., 2015; Martín‐López et al., 2009). For example, in the situation we presented, racial bias toward conservation messengers affected support for a conservation action (relocating people to protect lions) that would have serious human rights and livelihood consequences for local communities. As such, this study builds on existing knowledge of racism in conservation (Chaudhury & Colla, 2020; Rudd et al., 2021; Taylor, 2014) by highlighting the links between public perceptions of expertise and conservation's history of oppression, entrenched discrimination, and colonial narratives.

When considering participants’ social identities, the extent of support for animal rights had the biggest impact on responses across all three research questions. Namely, greater support for animal rights increased the perceived credibility of information in the post, the extent of support for relocating people to protect lions, and the perceived trustworthiness of the messenger. A similar pattern was observed across all questions for participants who reported that they would prioritize wild animals over people if their interests clashed. Given that the embedded message was heavily skewed toward an animal‐centered as opposed to a people‐centered solution to human–wildlife conflict (Appendix S3), these patterns were expected. The magnitude of these effects suggests orientations around animal rights support have significant potential to influence how the public views not only conservation strategies but also the credibility of those discussing conservation on X (Manfredo et al., 2020). Other research has shown comparable trends when examining the relationship between individuals’ social identities and their perspectives on contentious conservation scenarios (Bruskotter et al., 2019; Hare et al., 2024; Lute et al., 2014). It is therefore important that the potential for inherent beliefs to overshadow objectivity is considered, especially when public opinion on conservation issues could influence decision‐making and the direction of funds (Evans et al., 2023).

It is harder to interpret the pattern between participants’ support for human rights, the credibility they attached to the suggestion of relocating people to protect lions, and their inclination to support doing so. Only a small proportion of respondents did not support human rights (*n* = 17, *somewhat disagree* to *strongly disagree*). If we only consider participants who did support human rights, we see a trend that increasing agreement (from neutral to strongly agree) resulted in a decrease in perceived credibility and support for their recommendations (Figures 2b & 3b). This is as expected given that the post advocates for a strategy that would infringe on both human and land rights, highlighting the importance of accounting for nuances in individuals’ beliefs when considering their interactions with conservation information. When confronted with complex and controversial conservation problems, social identities can affect public opinion in multiple scenarios (Hare et al., 2024; Schroeder et al., 2021; Van Eeden et al., 2019, 2020). Our results add to these findings and highlight the need to consider not only how identity politics can influence public opinion of conservation problems, but also those sharing conservation messages.

Participants who were less trusting of scientists to act in the best interest of the public expressed weaker agreement across all research questions, suggesting general mistrust of scientists can influence perceptions of conservationists regardless of their expertise. We chose to ask this question broadly, despite scientists being a nonhomogeneous group, to facilitate the evaluation of an overall measure of trust. The concept of trust in scientists is a complex area of research (Hendriks et al., 2016), and our results echo others that advocate for the consideration of these dynamics when engaging the public with science, particularly in concert with individuals’ political ideology (McCright et al., 2013; Turel & Osatuyi, 2021). Additionally, participants who used X more frequently were more agreeable across all research questions. There is likely a reduced skepticism of conservation information and those sharing such messages on X that comes with increased use of the platform. It should again be noted that this study was conducted before Twitter was rebranded as X and lost a lot of scientific users (Hiltzik, 2023), so this shift should not have affected our results.

We restricted the identity factors and levels examined to those most intuitively relevant to the context of the study, in order to avoid statistically uninterpretable results. However, there are numerous other identity biases both within conservation and more broadly in society (e.g., regarding sexuality, religion, or disability), which would be valuable to explore in future studies. A deeper examination of geographic biases, particularly regarding racial stereotypes, nationality, and the contextual location of the conservation situation presented, would also be of interest. As an example, comparing these results to a study contextualized within a British conservation problem (e.g., strategies to reduce human–badger conflict [Cassidy, 2012]) may clarify queries around the potential effects of racial stereotyping as a proxy for perceived local knowledge of conservation issues. Furthermore, testing identity biases of publics outside the United Kingdom could provide additional insights for comparison and may influence the effects of the gender and race of the conservation messenger, dependent on contextual dynamics within the country. Examining these dynamics among those working within conservation advocacy groups and funding bodies could also prove interesting, as we might expect they would place more value on an individual's expertise than the public. In addition, it would be valuable for future studies to consider varying the content of the message itself. Specifically, testing controversial messages (such as permitting trophy hunting [Appendix S3.4]) against messengers we found to generate high versus low public trust would facilitate an understanding of whether stances on particularly contentious topics can sway public opinion of a conservation messenger. This would be of particular interest given recent studies have found both message framing and respondents’ characteristics to influence opinions on contentious issues in conservation (Frater et al., 2025; Niemiec et al., 2020).

Overall, this research shows that bias against conservation messengers based on their identity exists, and it manifests along complex, intersectional lines. We further found subtle differences in the impact of identity on each of the metrics—credibility, support, and trustworthiness—we tested (Appendix S8). In all cases, participants’ identities and social beliefs also influenced their responses. Men were perceived to be communicating a more credible lion conservation strategy than women, but gender had no effect on support for implementing that strategy. Instead, it was the interaction between the messenger's race and expertise that was relevant. White professors generated more support for relocating people to protect lions than White enthusiasts or field assistants. Conversely, Black professors generated less support than Black enthusiasts or field assistants. Finally, explicit trust in the messenger was affected by the interaction between the messenger's gender and race, as well as their expertise and race. Specifically, White professors were markedly more trusted than any other messenger, whereas Black women were the least trusted—reflecting deep‐rooted societal patterns of sexism and racism.

Our results demonstrate that public interaction with conservation information is not objective and is influenced by inherent biases, as has been observed in several other fields (Boling & Walker, 2021; Chávez & Mitchell, 2020; MacNell et al., 2015). Perhaps most alarming is the finding that in these hypothetical examples, the voices of experts in the field (professors and field assistants in this study) are viewed differently by virtue of their race, following long‐documented past and present patterns of discrimination and oppression within conservation (Chaudhury & Colla, 2020; Rudd et al., 2021). Not only does this suggest the potential for real‐world impacts on conservation outcomes based on identity bias, but it also raises questions about the dangers of homogenous ideas proliferating within conservation spaces owing to the reduced diversity of voices being heard (Hofstra et al., 2020). Although we found that respondents’ social identities and beliefs had the strongest influence on all outcomes, even when accounting for these, the identity of the messenger mattered.

Despite increased attention toward these issues within the field of conservation over recent years, action is still limited and slow. Our results add further fuel to the drive toward addressing these injustices and specifically call for increased communication about such biases within conservation with wider stakeholders, such as the public, decision makers, politicians, and funders. Conservation as a field relies upon vast and varied groups, all of whom carry their own set of biases and preconceived ideas about who a conservation expert is. Although these results could be used to advocate for manipulating conservationists’ identity factors advantageously when communicating recommendations, the ethics of this are highly dubious. Instead, we argue that it is only by collectively taking responsibility for ensuring equity, inclusivity, and platforming diverse voices that such biases and misconceptions can be rectified. This will ultimately build a more just, equitable, and effective conservation arena (see Appendix S9 for extended results and discussion).

## AUTHOR CONTRIBUTIONS

**Lauren F. Rudd**: Conceptualization; methodology; formal analysis; investigation; writing—original draft; funding acquisition. **Yolanda Mutinhima**: Conceptualization; writing—review and editing. **Shorna Allred**: Conceptualization; writing—review and editing. **Amy J. Dickman**: Conceptualization; writing—review and editing; supervision. **Darragh Hare**: Conceptualization; methodology; writing—review and editing; supervision.

## Supporting information



Supporting Information
