# Supplementary material for: How identity bias affects perceptions of conservation messages on social media
Source: Conserv Biol. 2026 May 8;40(4):e70315. doi: 10.1111/cobi.70315 (PMC13392792; doi:10.1111/cobi.70315)
Supplement: Supplementary file 1 — Supporting Information [file COBI-40-e70315-s001.docx]

# **Supporting information for “Identity bias and public perceptions in lion conservation”**

**Appendix S1- Full Questionnaire**

**SECTION 1- LANDING PAGE**

Please read through the information below about our study before deciding whether to participate. You may ask any questions before deciding to take part by contacting the principal researcher (details below).

By participating in this online survey about the credibility of lion conservation recommendations (it will take approximately 5-10 minutes), you will help us understand how people respond to information about lion conservation on Twitter. You do not need any specialist background knowledge to participate. Our results from this study might help to inform how conservationists frame messages about lion conservation.

We will store the responses you provide in a password-protected electronic file. We will not ask you to provide any information that would identify you and we will not store your IP address. Only researchers working directly on this study will have access to the information you provide.

The data you provide may be transferred to, stored and/or processed at a destination outside the UK and the European Economic Area ("EEA"). By submitting data, you agree to this transfer, storing or processing. We may use the information you provide in academic publications such as reports and journal articles, but we will only analyze and report responses in general terms.

Qualtrics LLC is the data controller with respect to your personal data and, as such, will determine how your personal data is used. Please see their privacy notice here [www.qualtrics.com/privacy-statement/]. Qualtrics LLC will share only de-identified data with the University of Oxford, for the purposes of research.

If you choose to participate, we will first ask some questions to determine whether you are eligible for the study.

You can choose to withdraw for any reason at any point during the study by closing your browser tab. You will receive payment for participation if you complete the study, which includes answering some required questions. Some parts of the questionnaire mention people and/or animals dying, but there are no pictures or graphic descriptions.

This project has been reviewed by, and received ethics clearance through, a subcommittee of the University of Oxford Central University Research Ethics Committee [reference number R79948/RE001].

The principal researcher is Lauren Rudd, who works in the Wildlife Conservation Research Unit in the Zoology Department at the University of Oxford. This study is being conducted in collaboration with other researchers at the University of Oxford and Cornell University.

If you have a concern about any aspect of this study, please email Lauren Rudd (lauren.rudd@gtc.ox.ac.uk) and we will do our best to answer your query. We will acknowledge your concern within 10 working days and give you an indication of how we will deal with it. If you remain unhappy or wish to make a formal complaint, please contact the Chair of the Medical Sciences Interdivisional Research Ethics Committee at the University of Oxford who will seek to resolve the matter as soon as possible: Email: ethics@medsci.ox.ac.uk; Address: Research Services, University of Oxford, Boundary Brook House, Churchill Drive, OX3 7GB.

By confirming you are 18 or older and selecting "Yes, I agree to take part" below, you indicate that you voluntarily agree to participate in this study.

Please note that you may only participate in this study only if you are 18 years of age or over.

☐ I certify that I am 18 years of age or over

If you have read the information above and agree to participate with the understanding that the data you submit will be processed accordingly, please check the relevant box below to get started.

☐ Yes, I agree to take part

*After providing consent, respondents will answer a few general questions to confirm eligibility and prevent oversampling from some demographics. This means we collect data only from eligible respondents, so we will not waste ineligible respondents’ time.*

| *Item number* | *Measuring* | *Item* | *Response options* |
| --- | --- | --- | --- |
| 1.1 | Gender Quota | Which best describes how you identify your gender? | · Woman  · Man  · Non-binary  · In another way  · Prefer not to say |
| 1.2 | Age quota | How old are you? | · 18-29  · 30-44  · 45-59  · 60 or older  · Prefer not to say |
| 1.3 | Ethnicity Quota | Which best describes how you identify your ethnicity? | White  · English/Welsh/Scottish/Northern Irish/British  · Irish  · Gypsy or Irish Traveler  · Any other White background  Asian/Asian British  · Indian  · Pakistani  · Bangladeshi  · Chinese  · Any other Asian background  Black/African/Caribbean/Black British  · African  · Caribbean  · Any other Black/African/Caribbean background  Mixed/Multiple ethnic groups  · White and Black Caribbean  · White and Black African  · White and Asian  · Any other Mixed/Multiple ethnic background  Other ethnic group  · Arab  · Any other ethnic group  · Prefer not to say |
| 1.4 | Twitter usage | Approximately how often have you used Twitter in the past 6 months? *This could involve posting your own content and/or reading content posted by others* | · Every day  · At least once a week  · At least once a month  · Less than once a month |

**SECTION 2: BACKGROUND/CONTEXT PAGE**

Please carefully read the information below, which describes the current situation surrounding lion conservation and management.

It is estimated that there are as few as 23,000 wild lions living in Africa today, around half as many as there were 20 years ago. Many lions live in protected areas such as national parks. As lions require large areas, they often move into land surrounding protected areas, where people live.

More than a quarter of the area where lions still exist is entirely outside of protected areas, alongside people. Many of the people that live alongside lions keep livestock such as goats and cows, which are essential to their livelihoods.

Some of the biggest threats to lions today are the loss of wild habitat and prey, and human-lion conflict, for example, when people legally or illegally kill lions to protect themselves or their livestock.

Moving “problem lions” that prey on livestock to a different place can sometimes reduce human-lion conflict in the original area. However, doing so can also increase human-lion conflict in the area where the lions are relocated, with risks for both people (who might suffer attacks) and lions (who might then be killed).

Lion conservation is expensive, and in most places, photo-tourism alone cannot cover the costs of effective conservation. Legal, regulated trophy hunting of lions can generate additional funding to incentivise conservation and may create local income and jobs.

Some people believe that trophy hunting lions is acceptable. However, others believe that killing lions, especially for recreation or trophies, is unacceptable.

When you have read this information, please click “Next”.

**SECTION 3: MAIN STUDY- CREDIBILITY OF CONSERVATION INFORMATION ON TWITTER**

*Each respondent was presented with one fictitious Twitter (now X) profile image as shown below, alongside the following questions.*


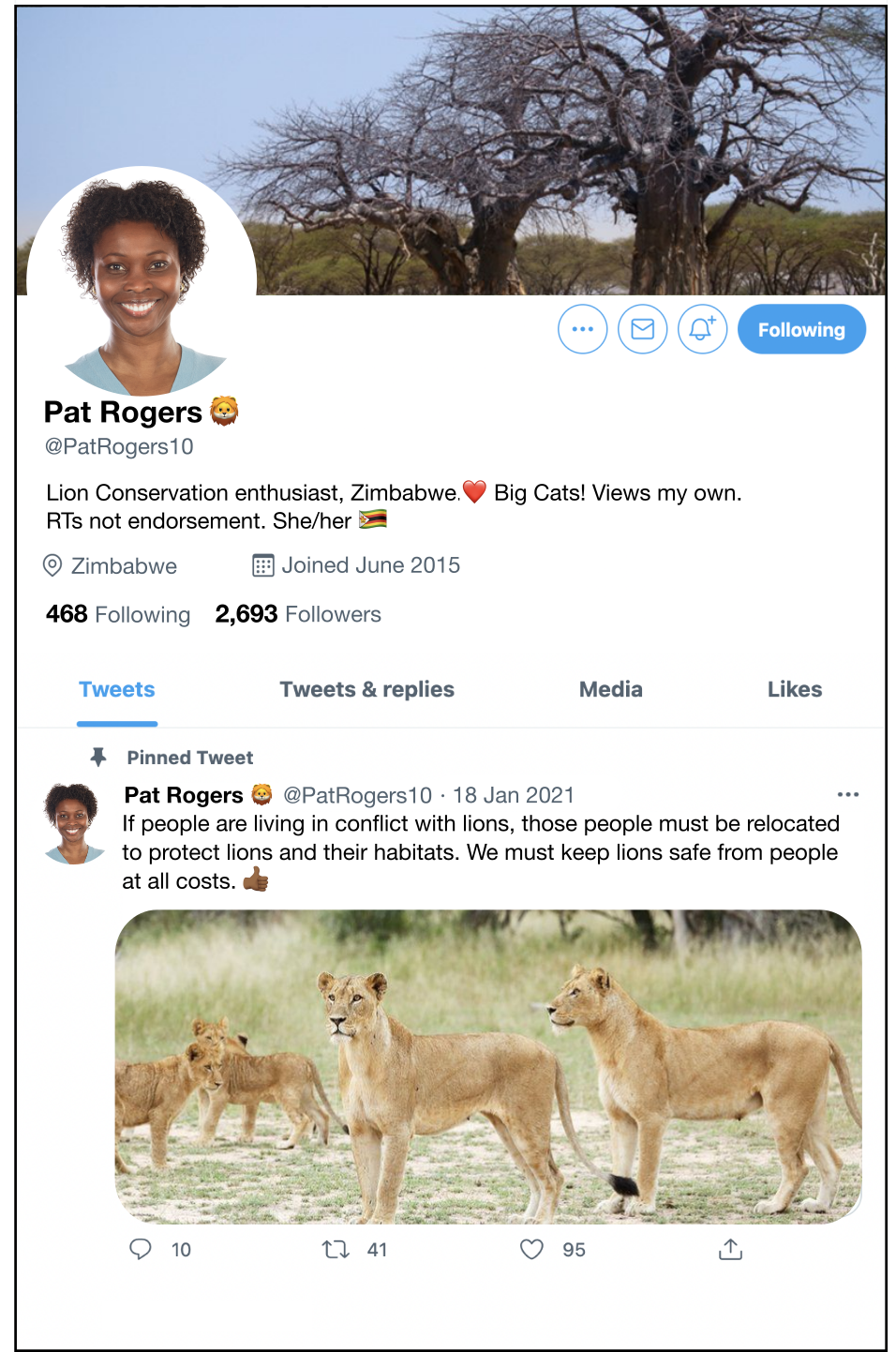

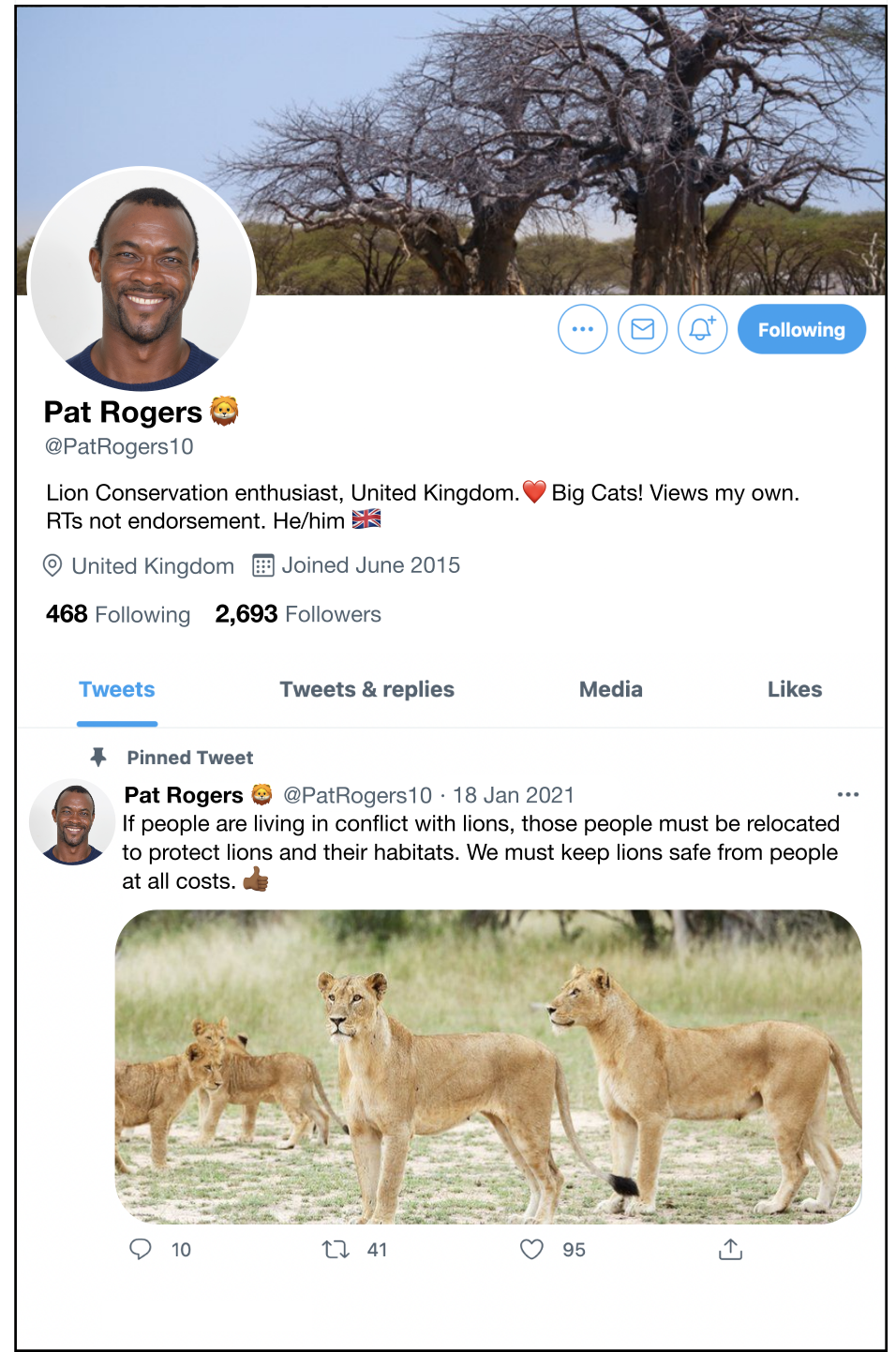

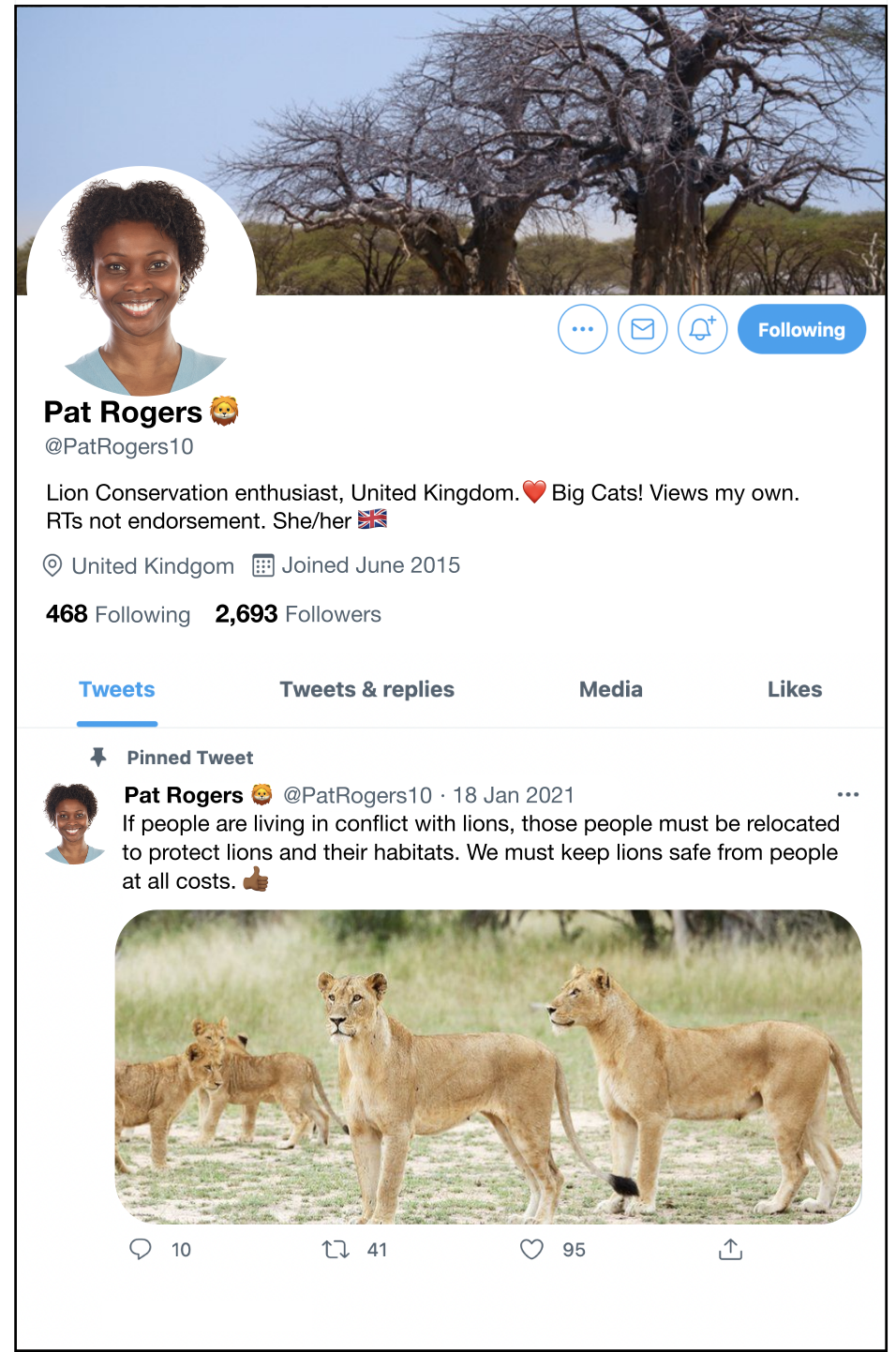

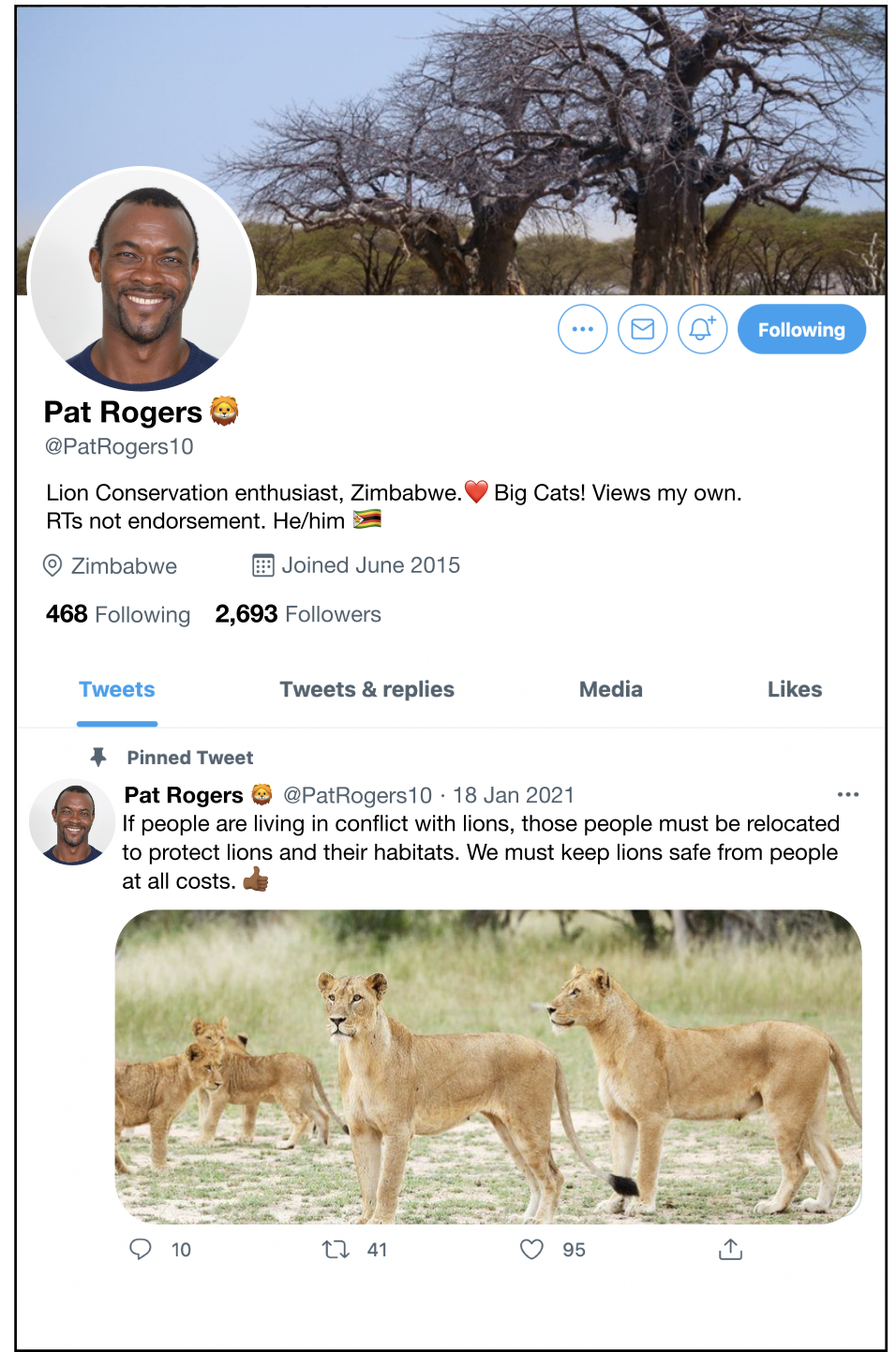


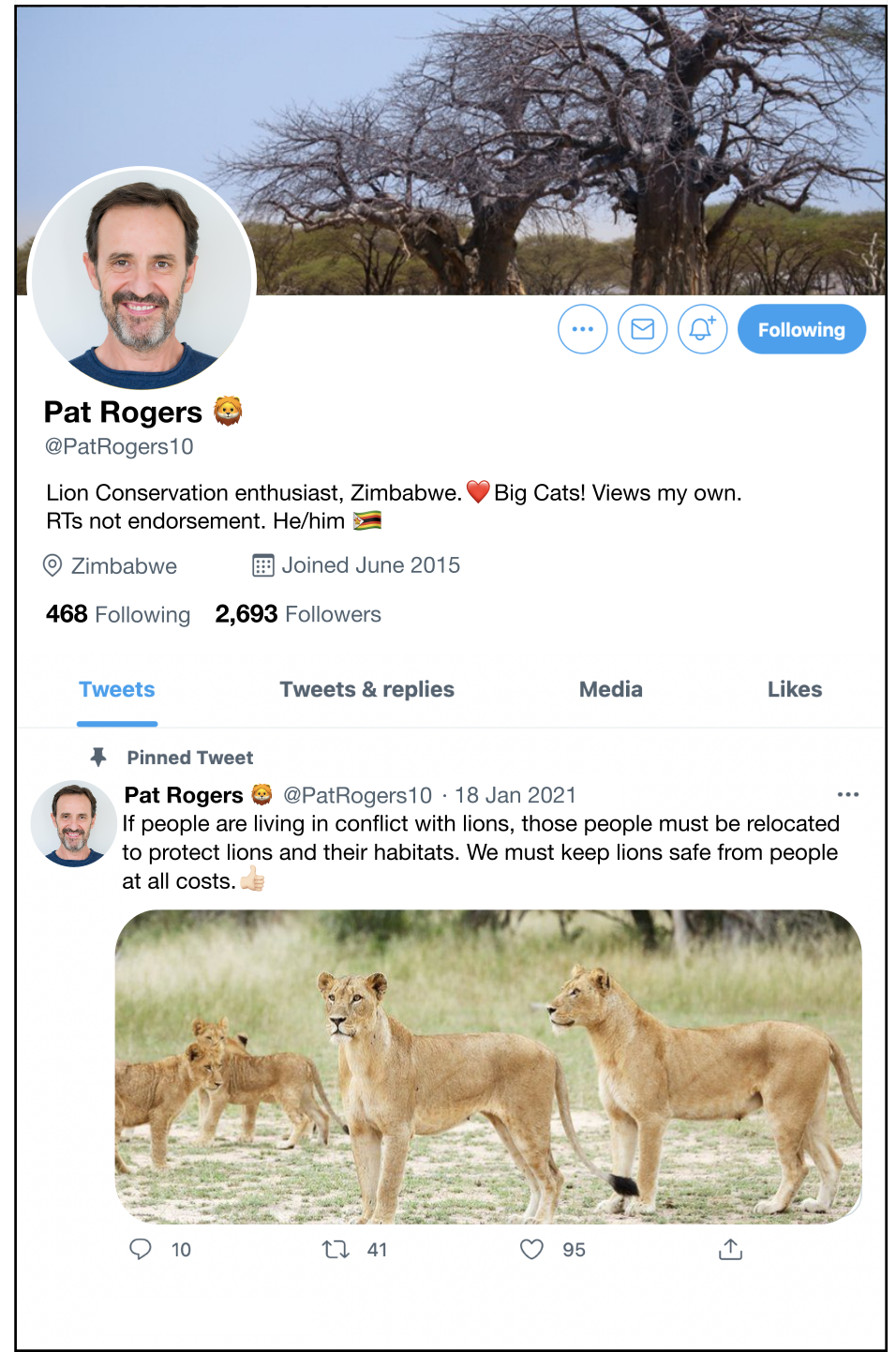

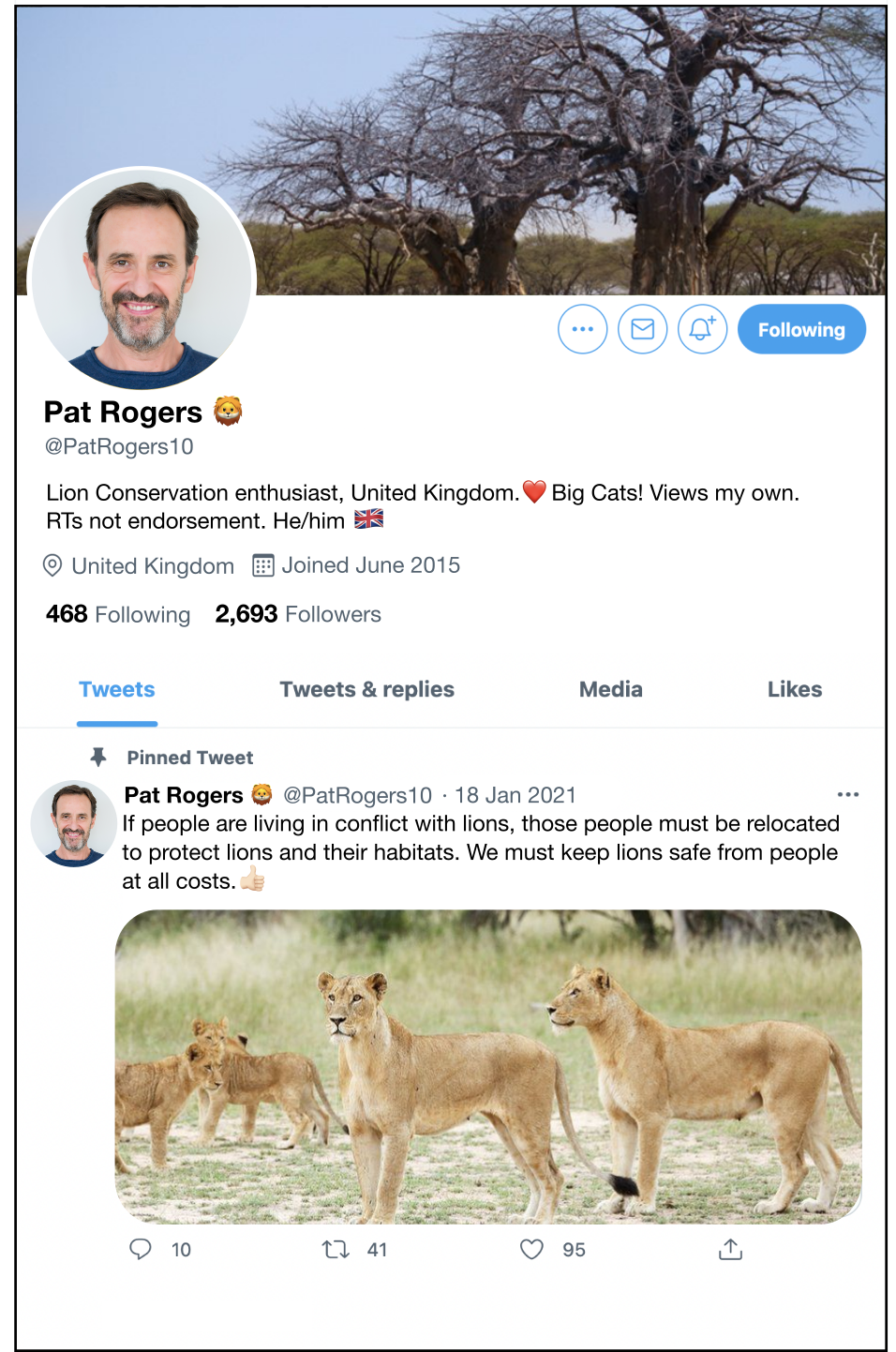

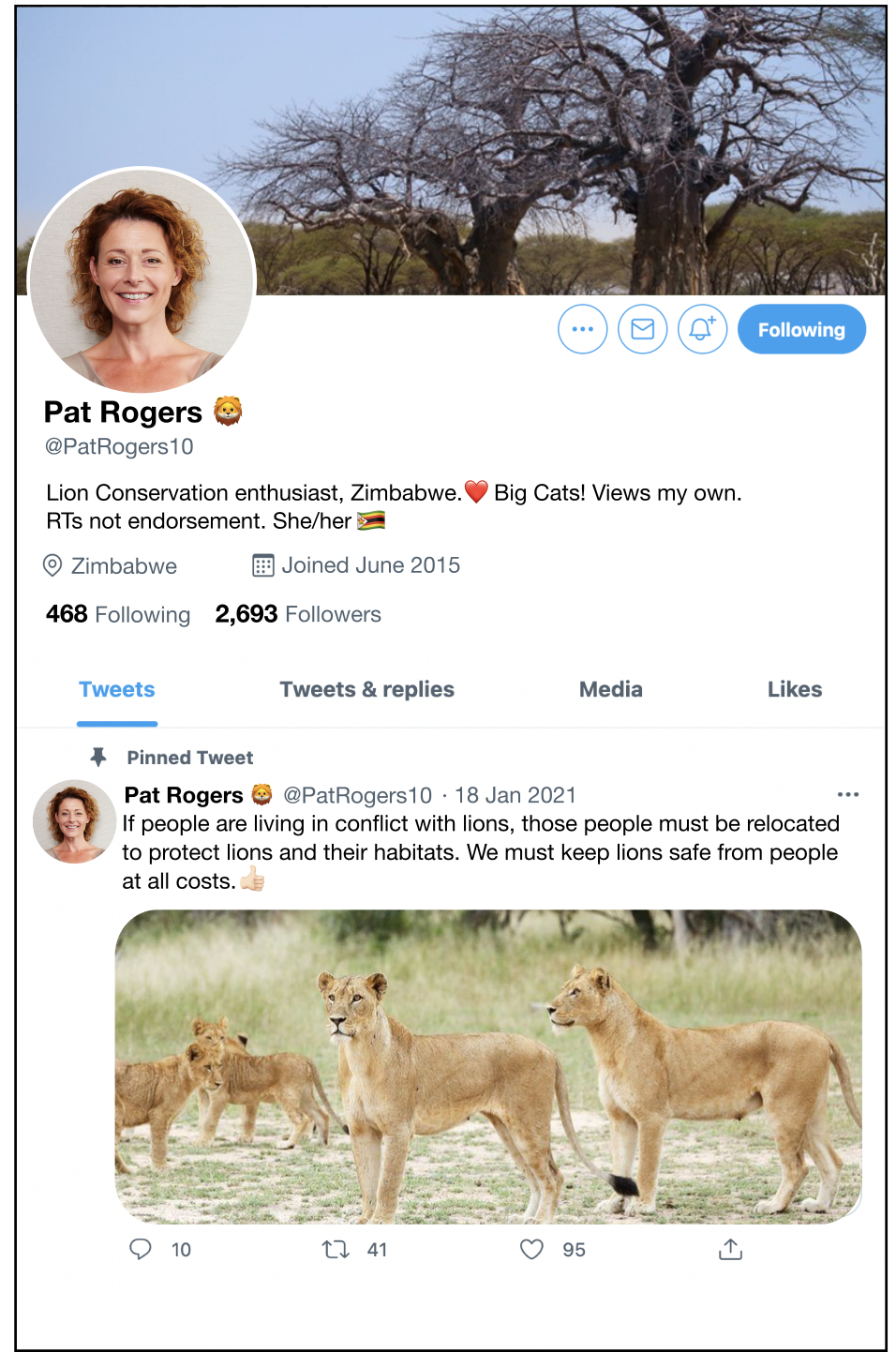

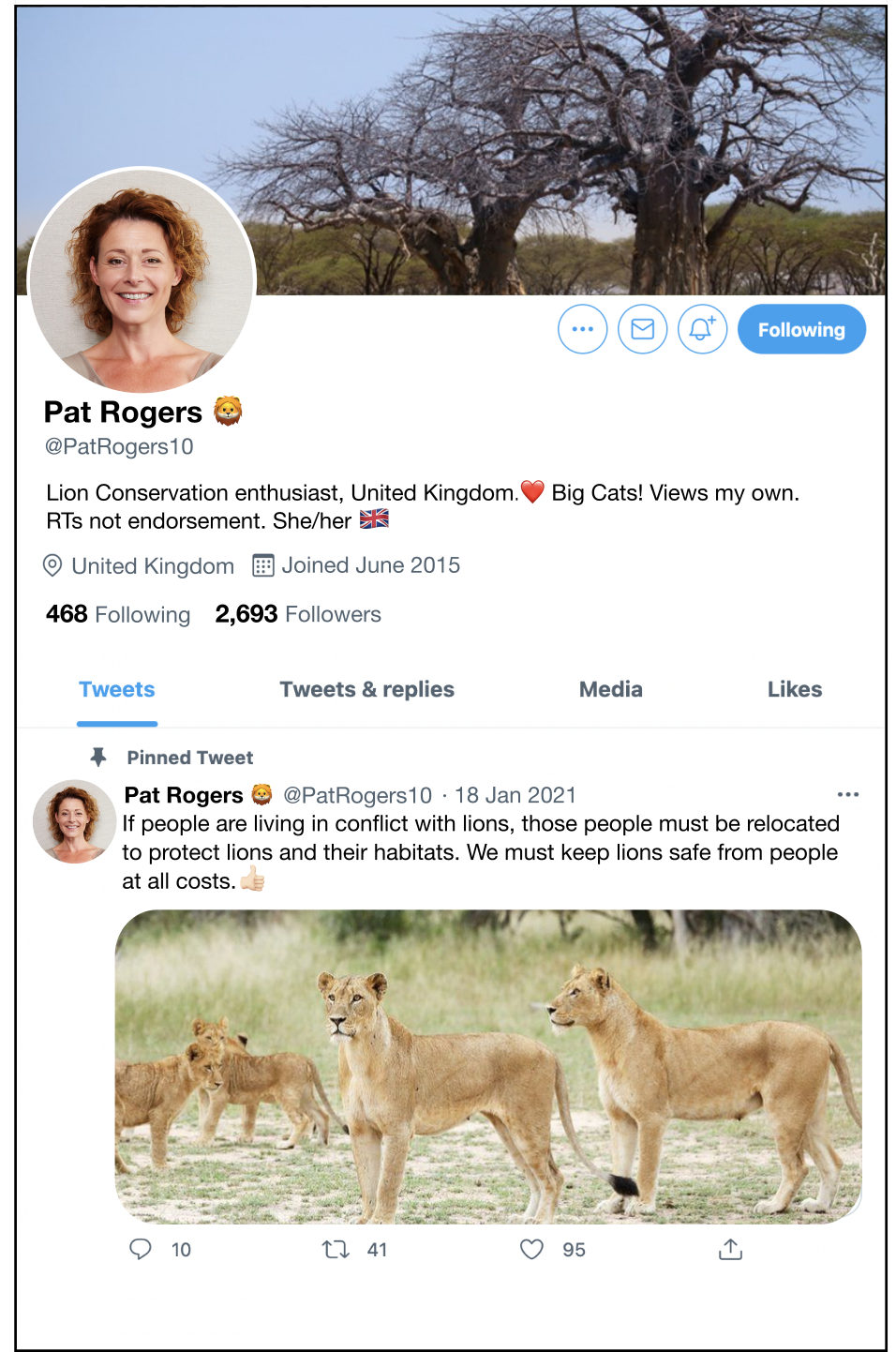


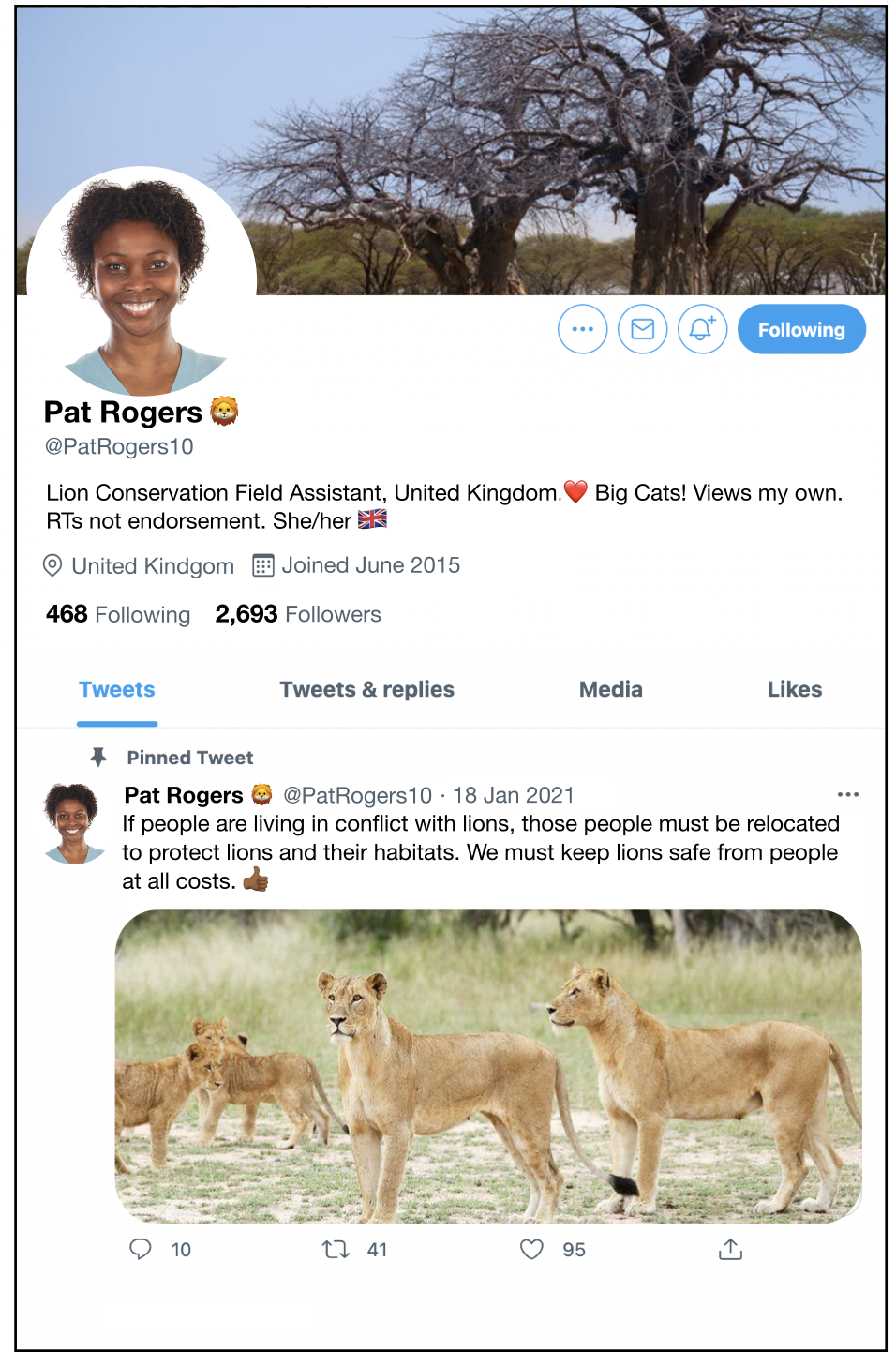

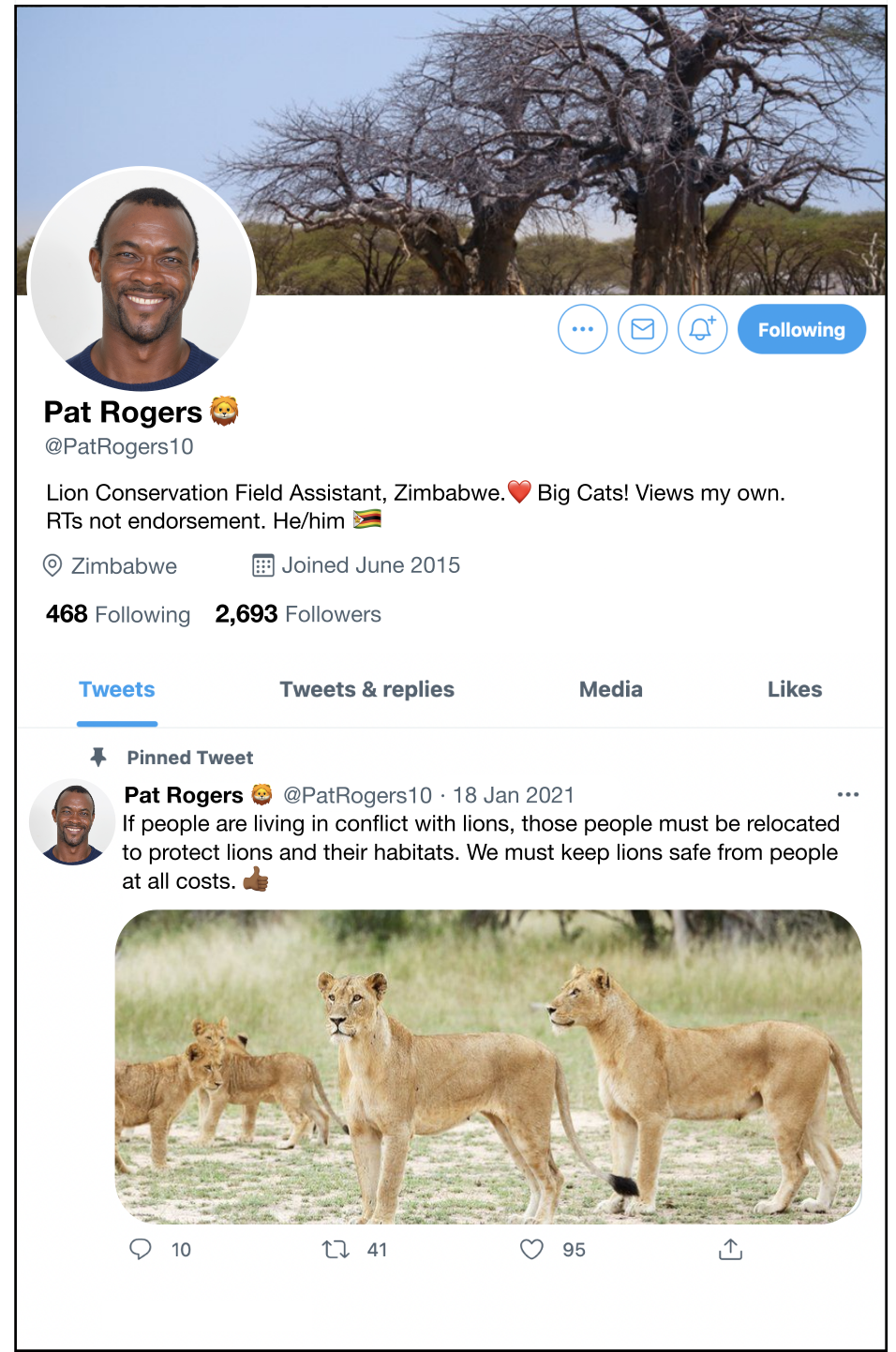

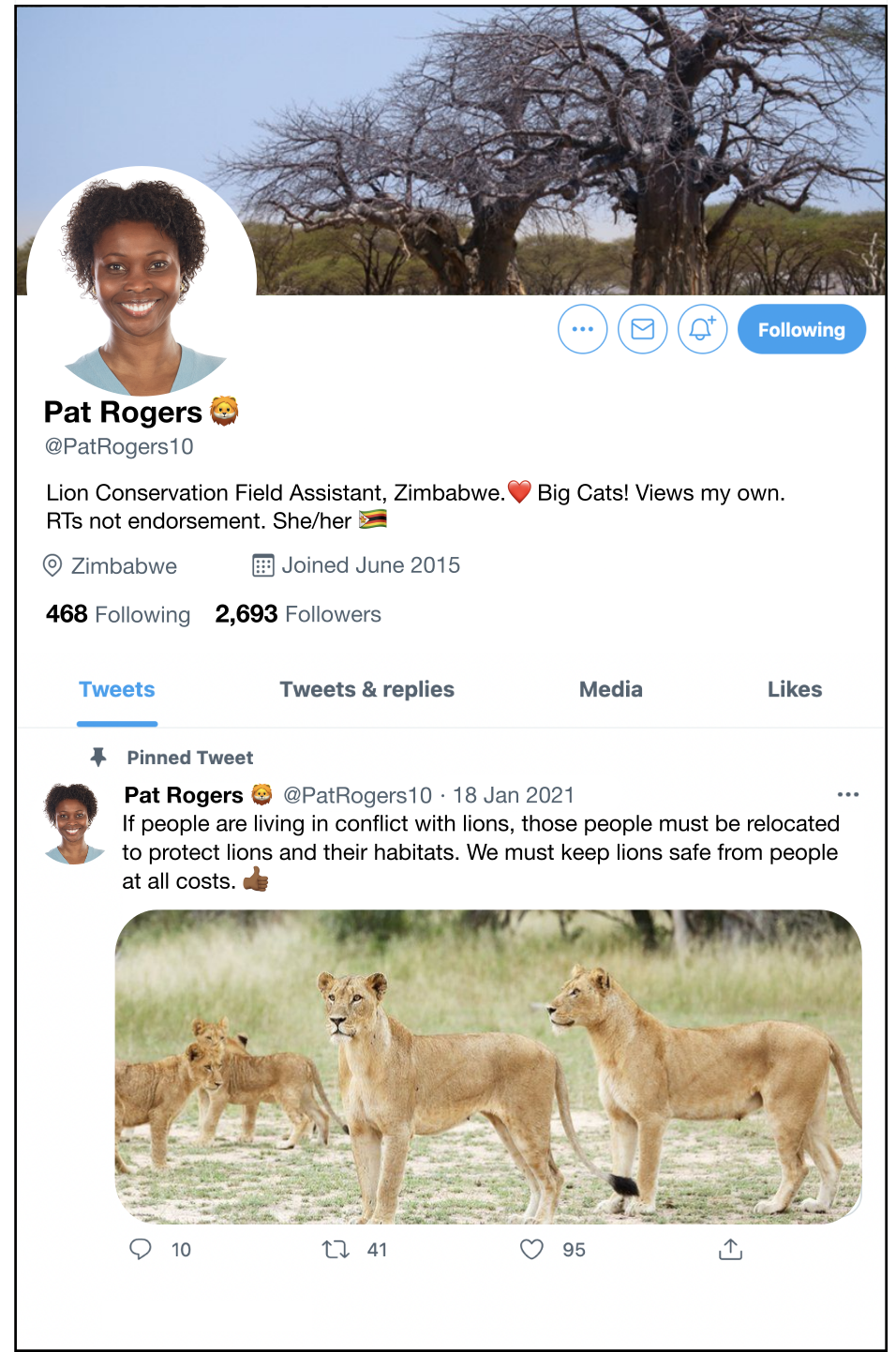

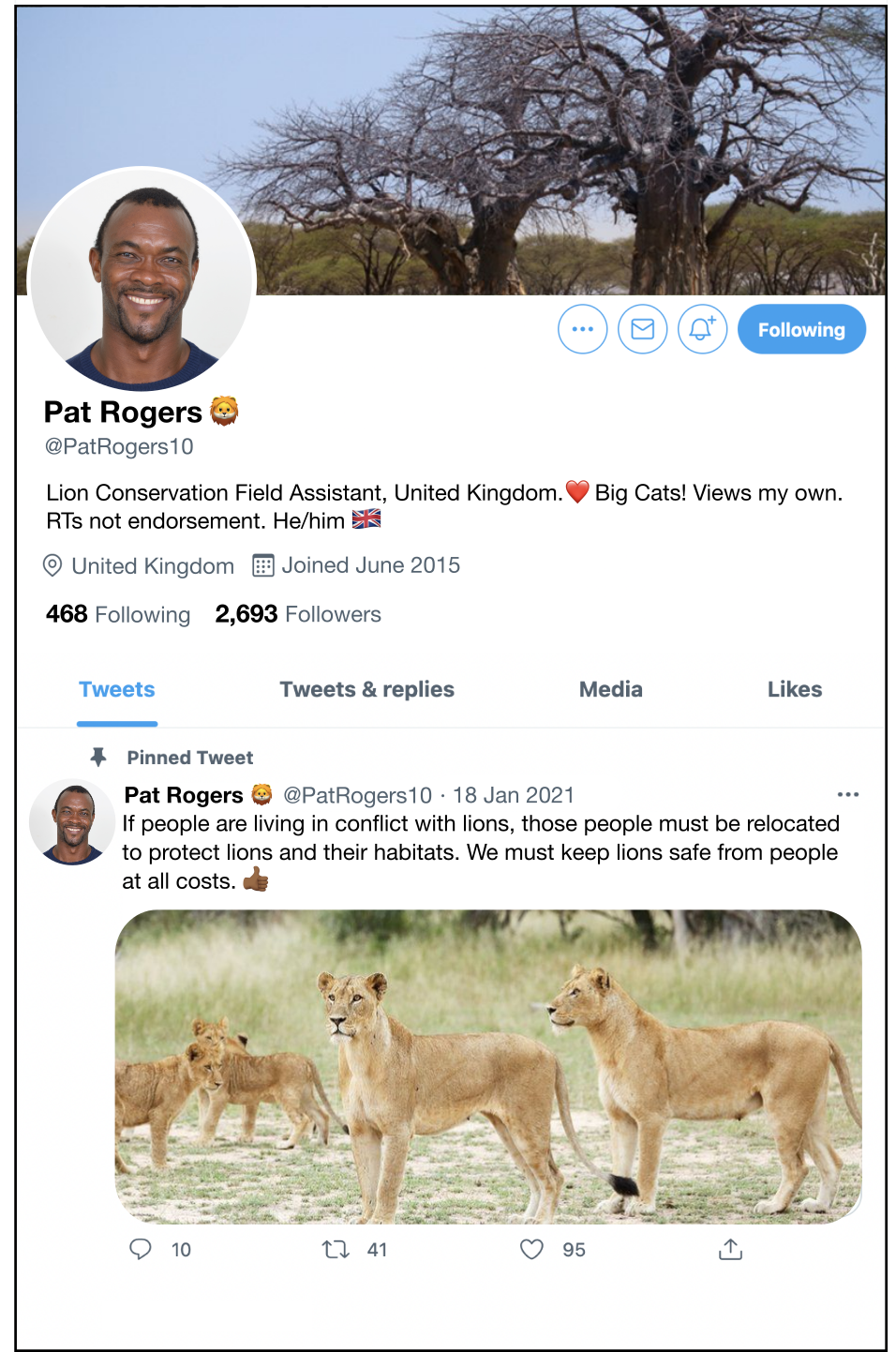


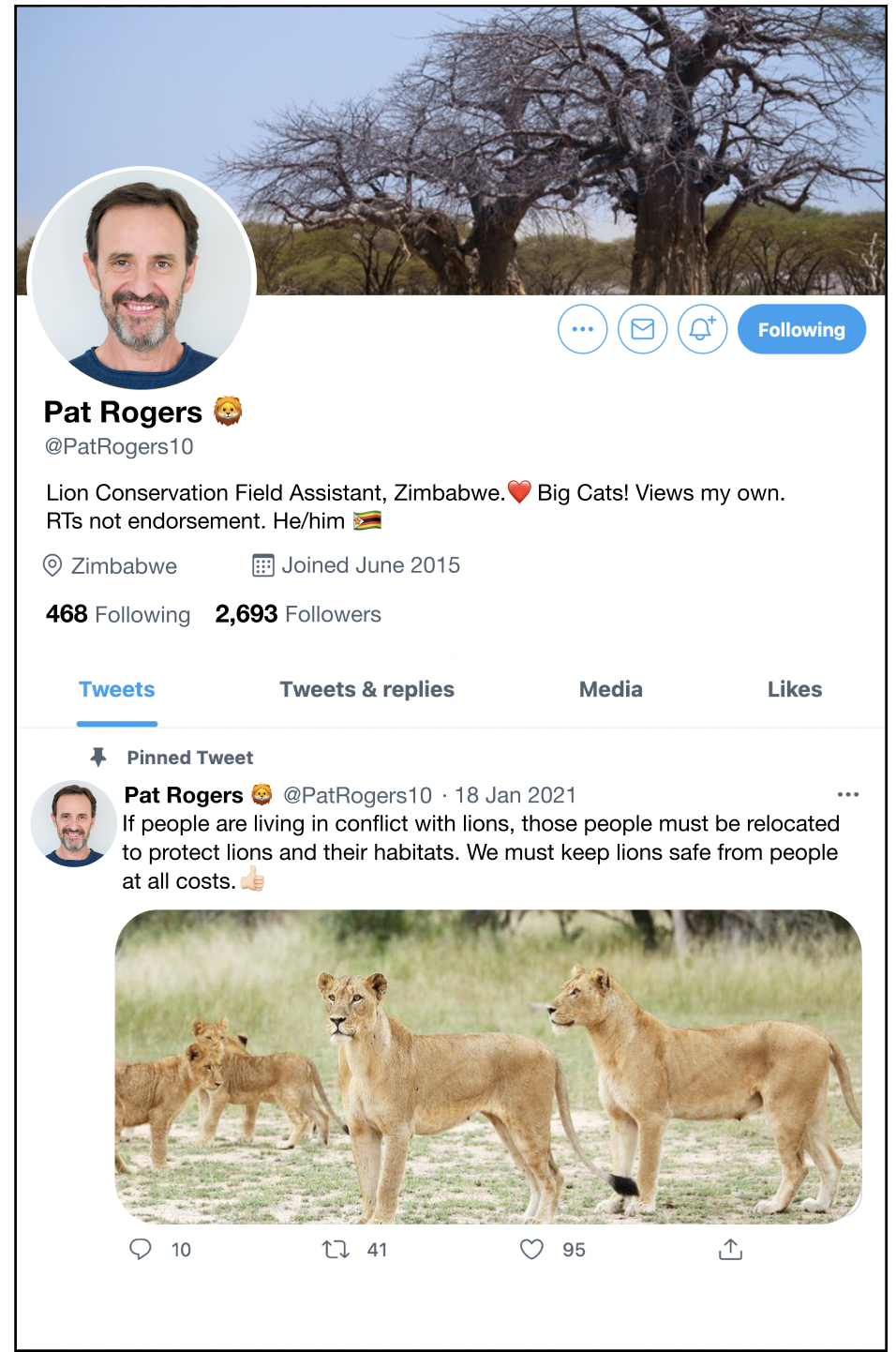

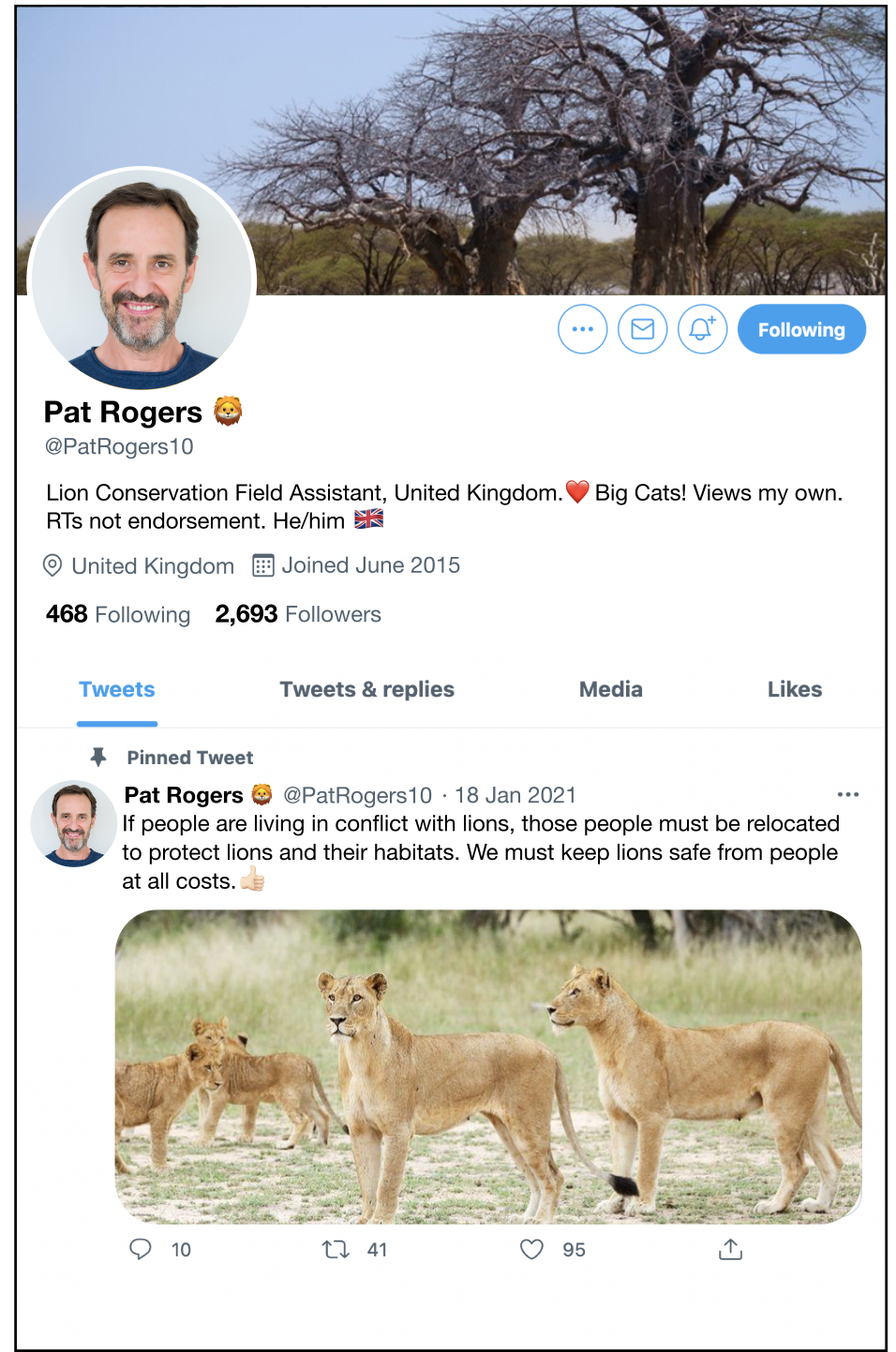

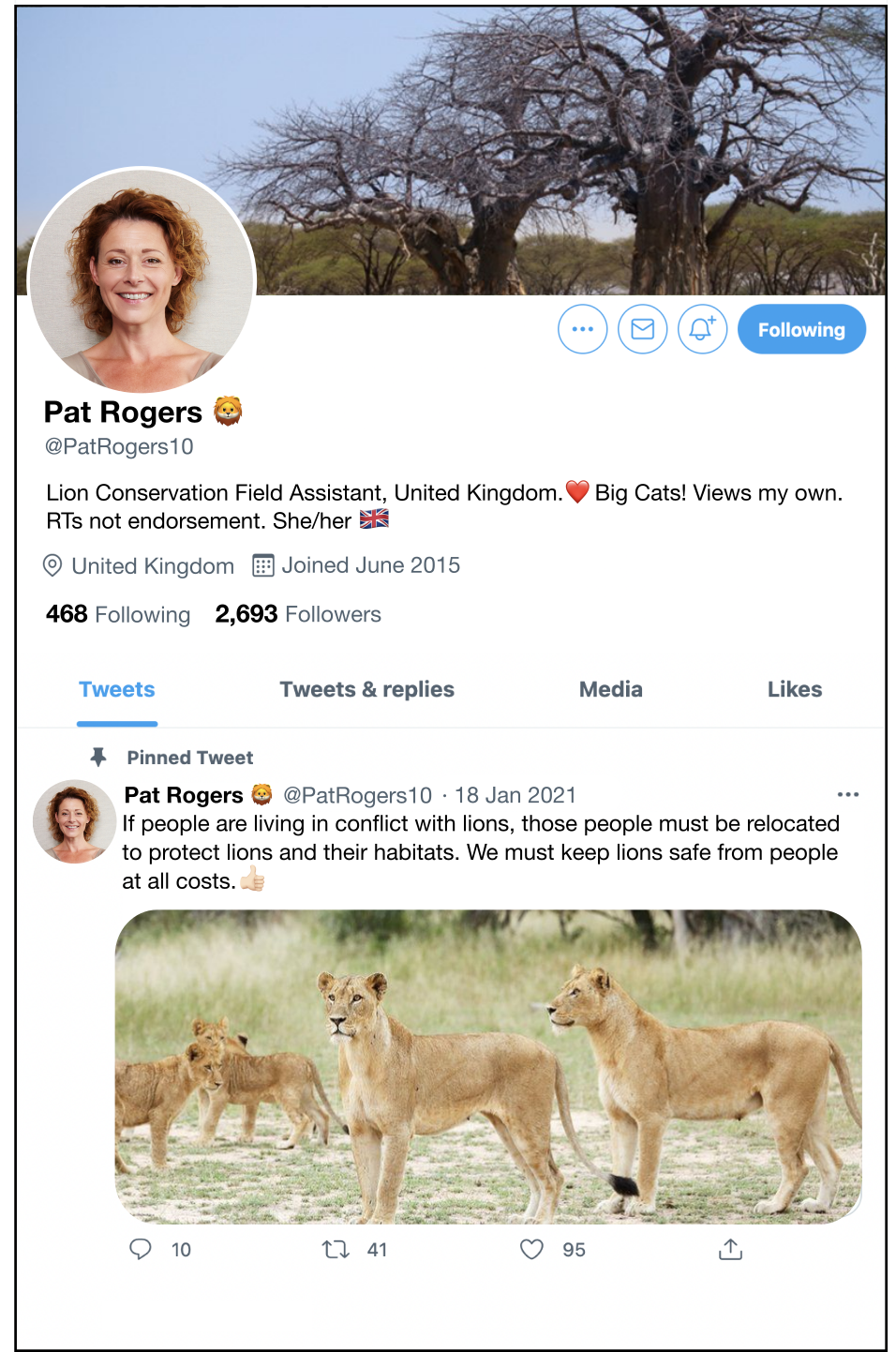

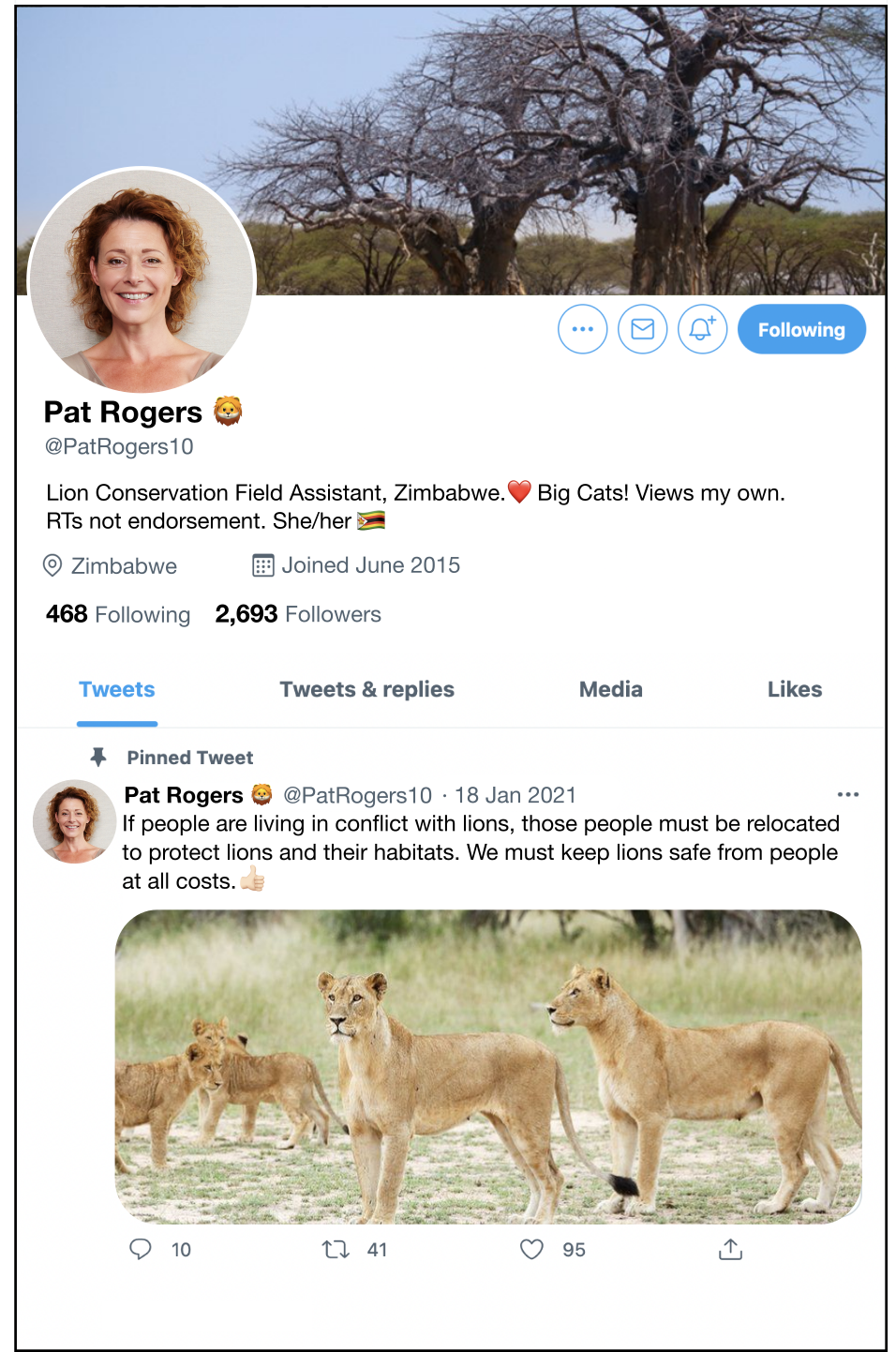


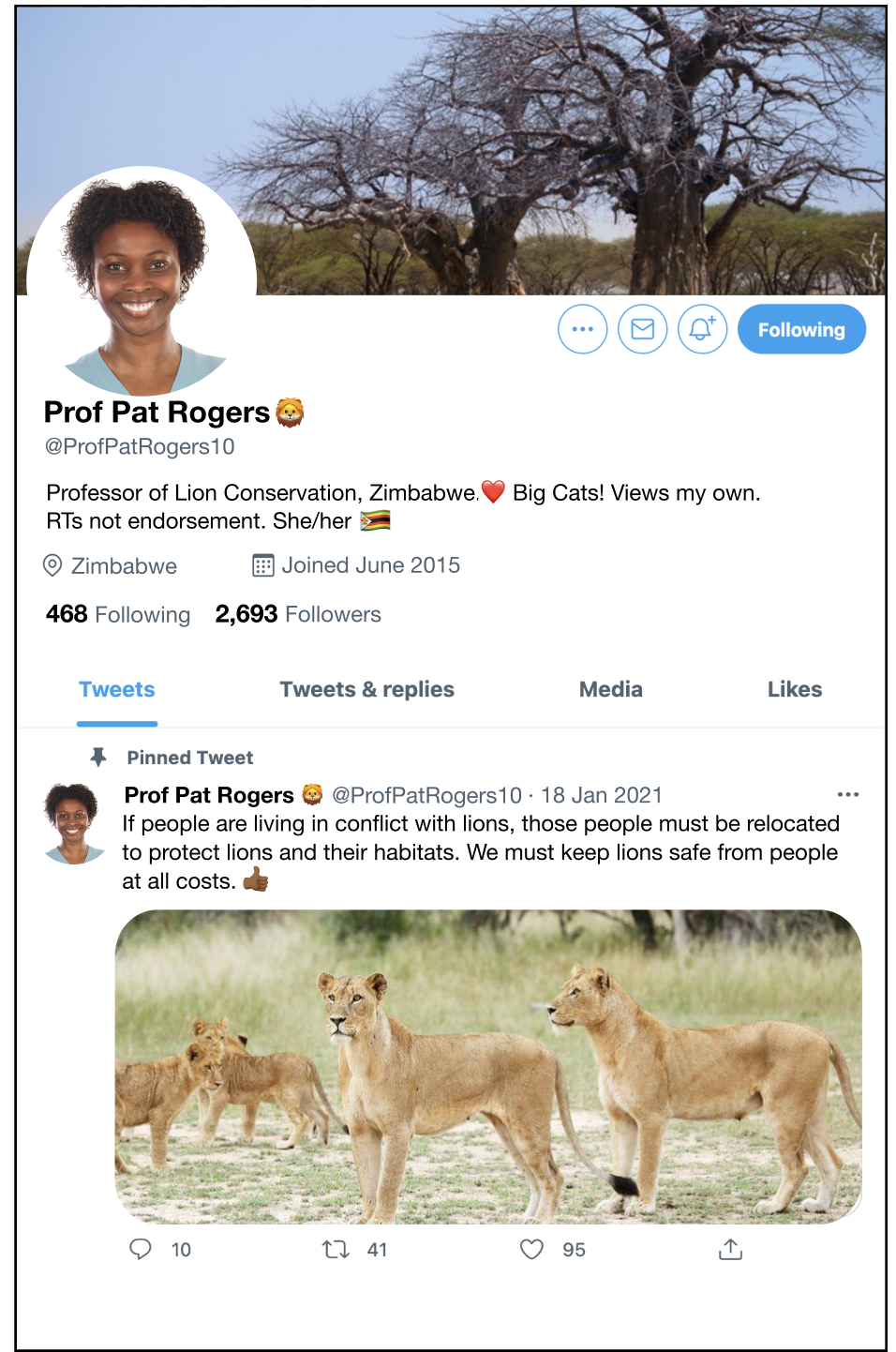

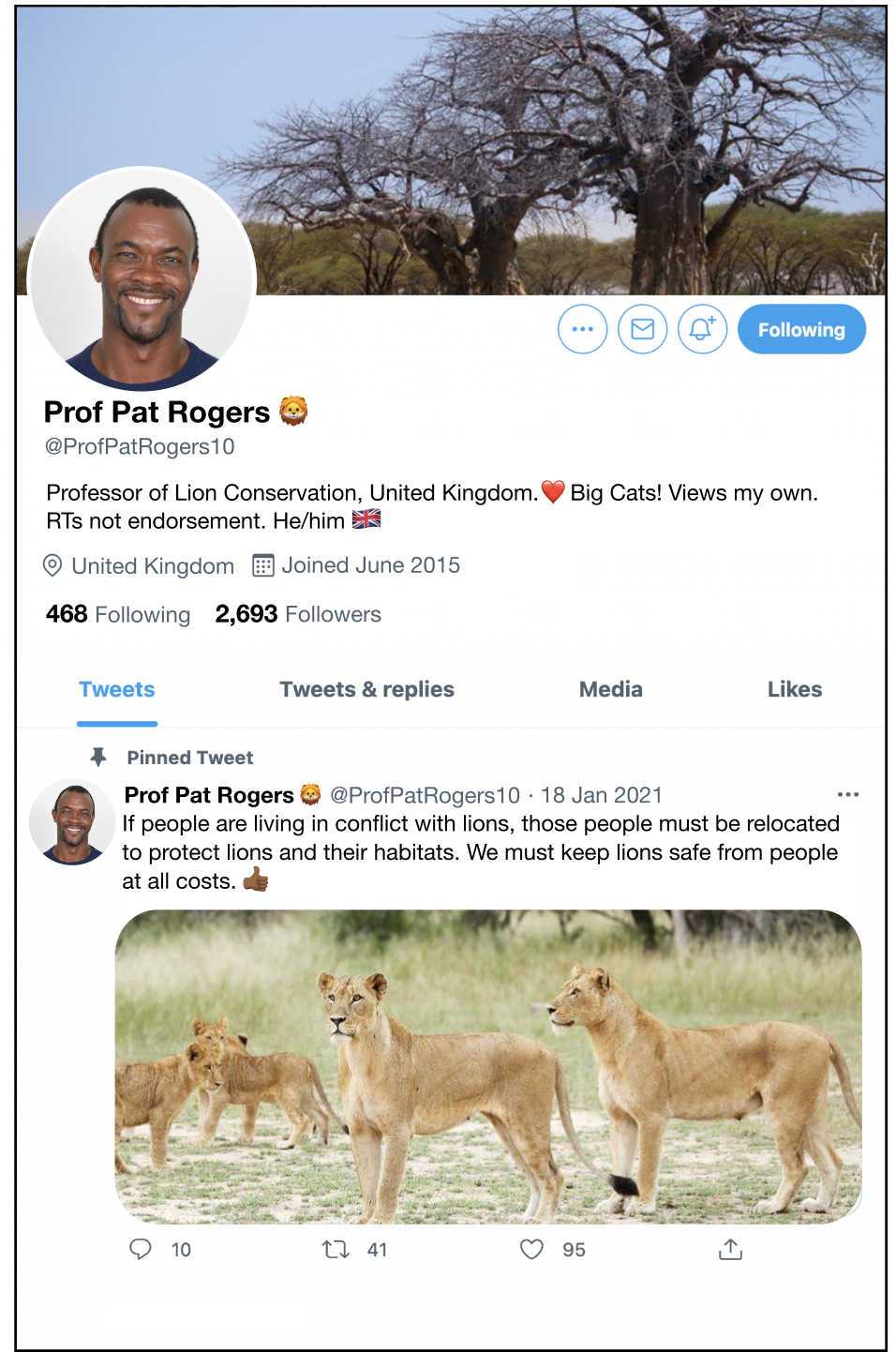

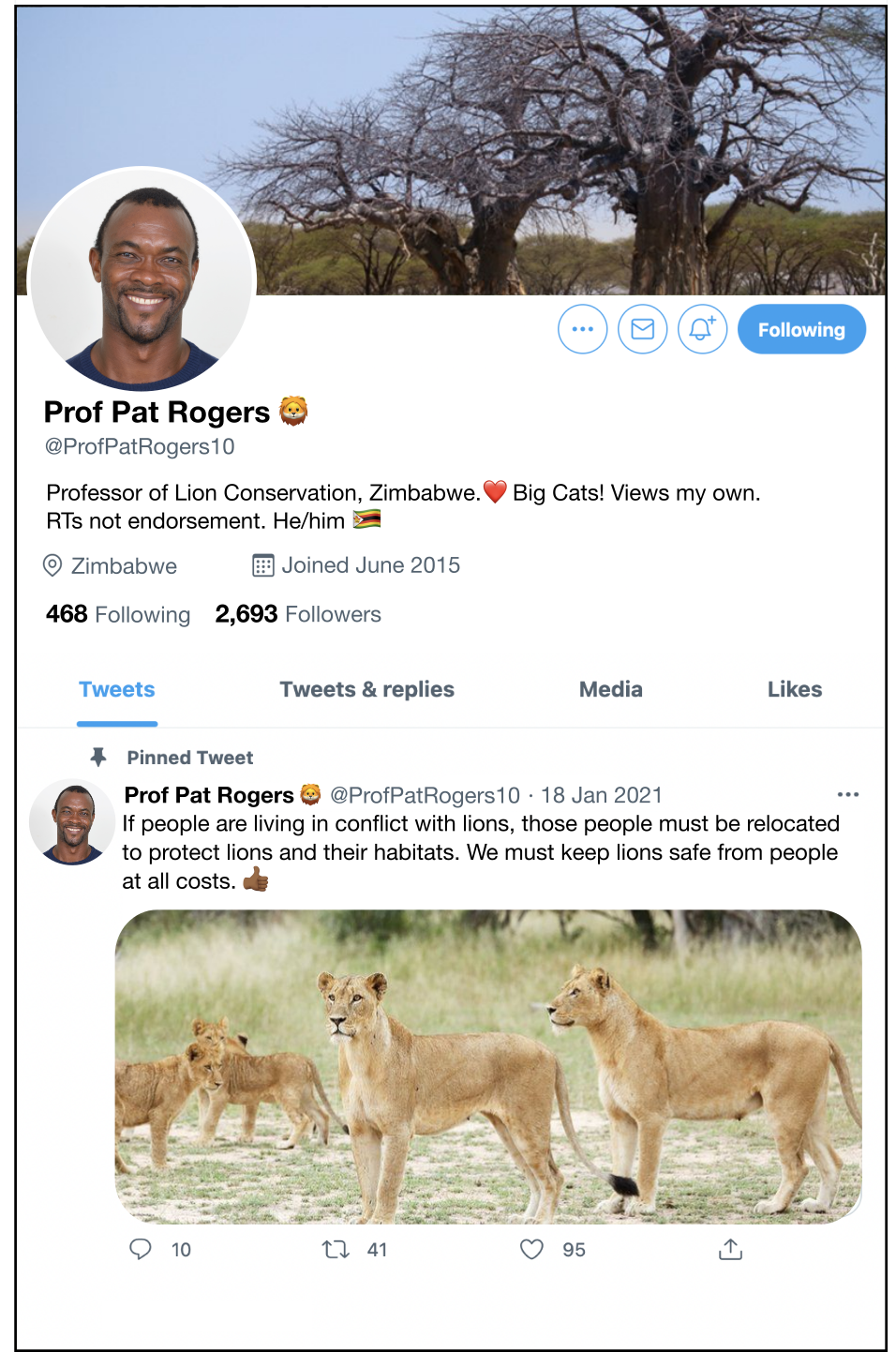

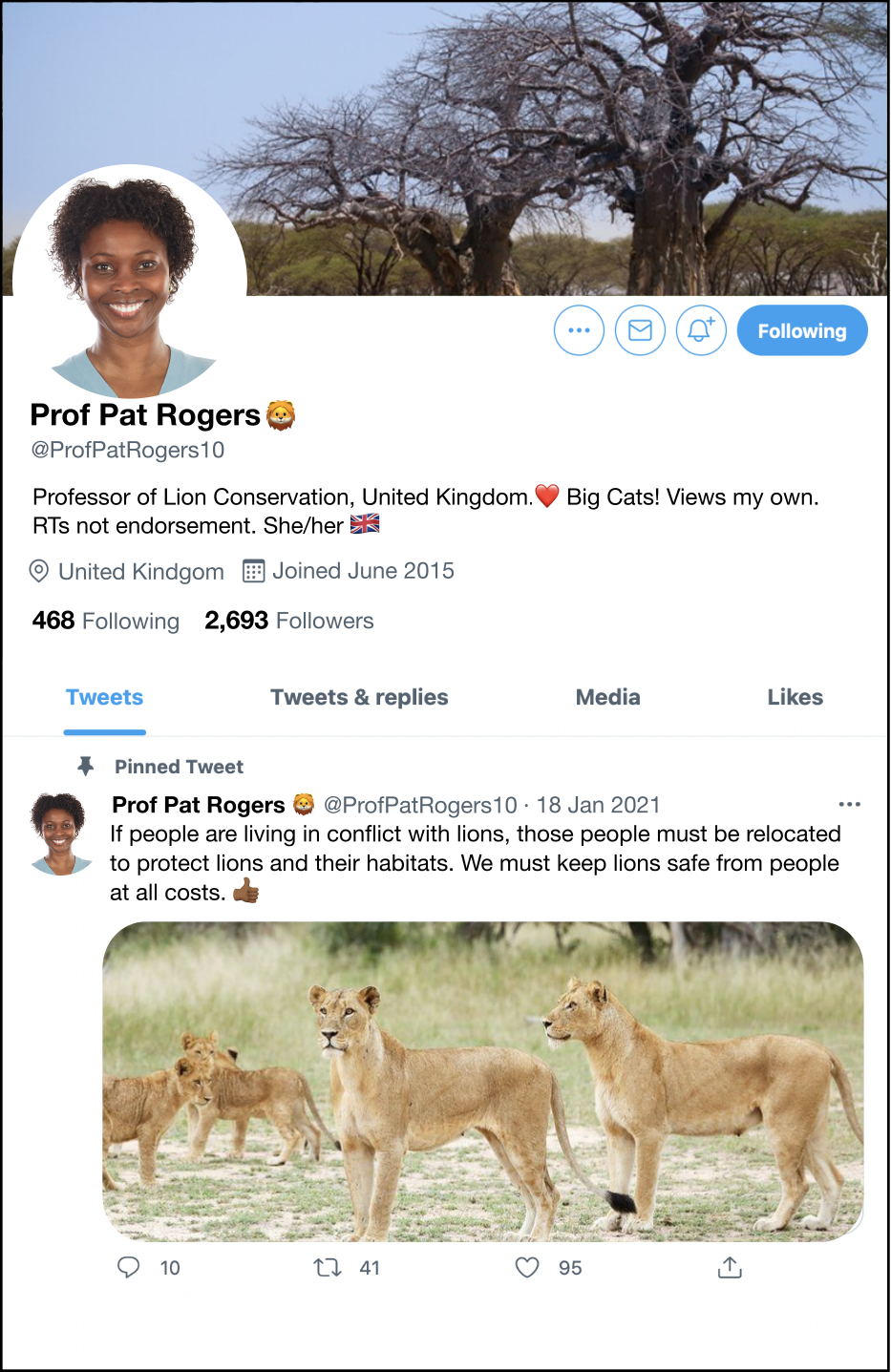


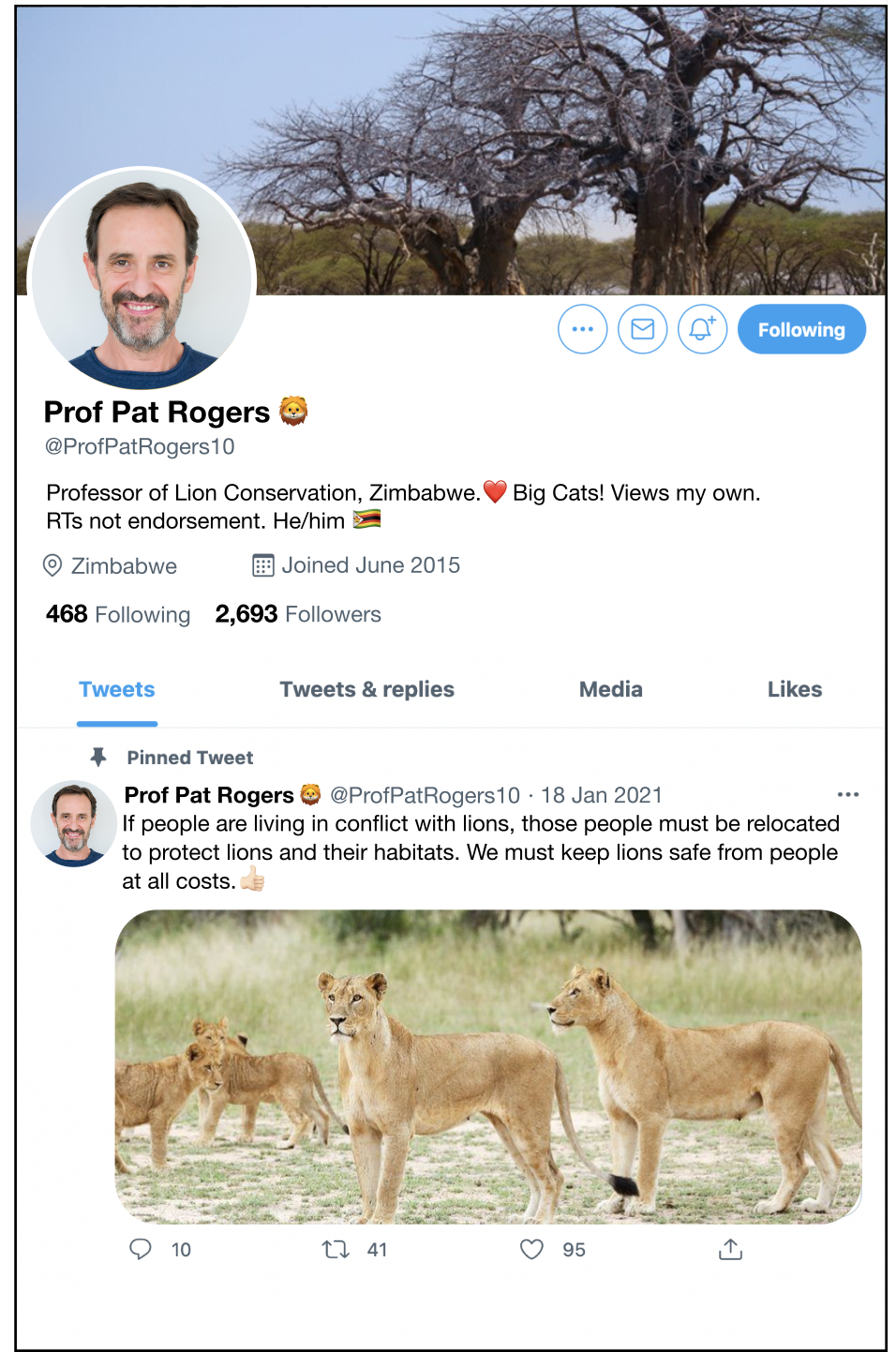

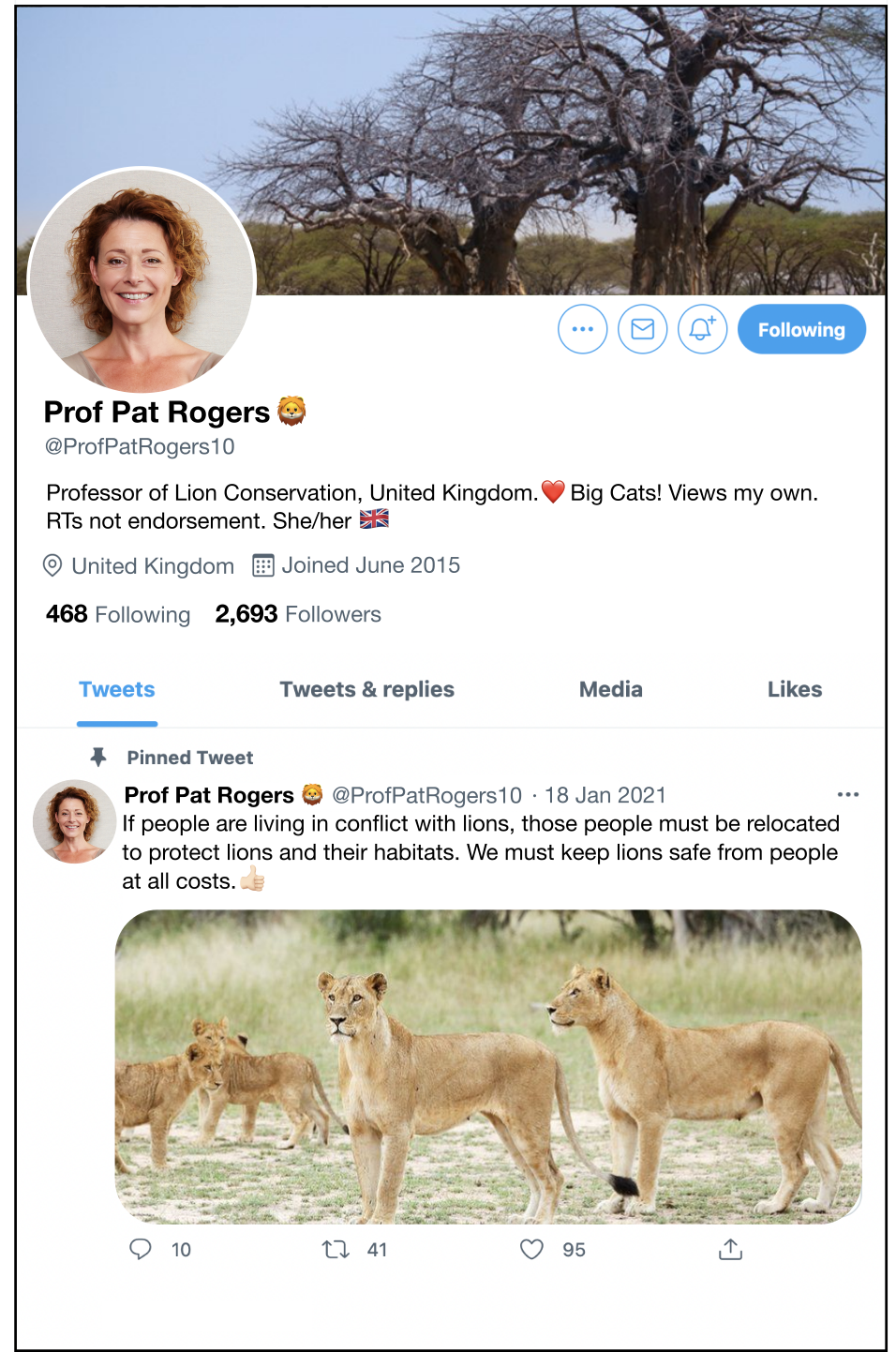

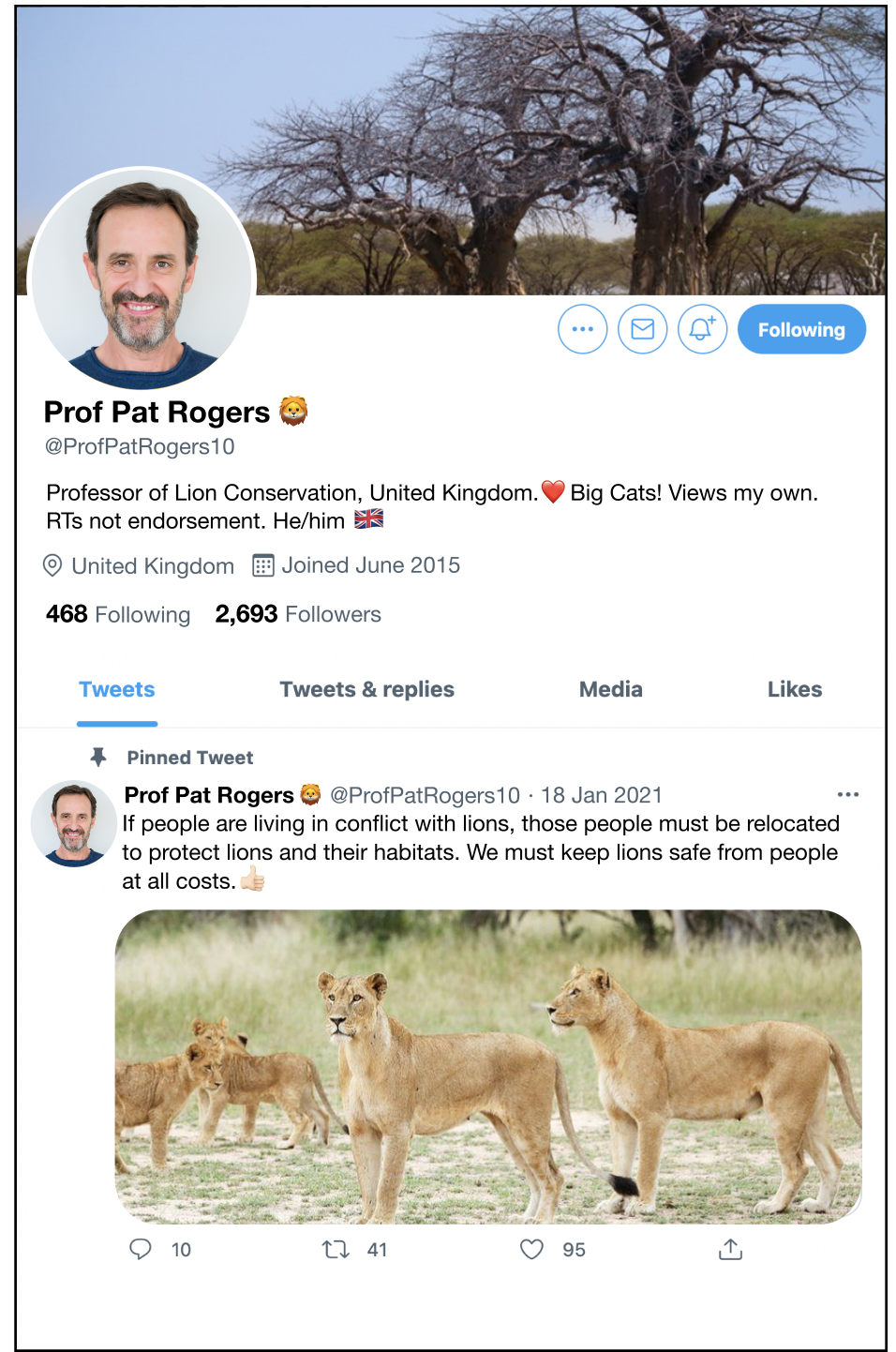

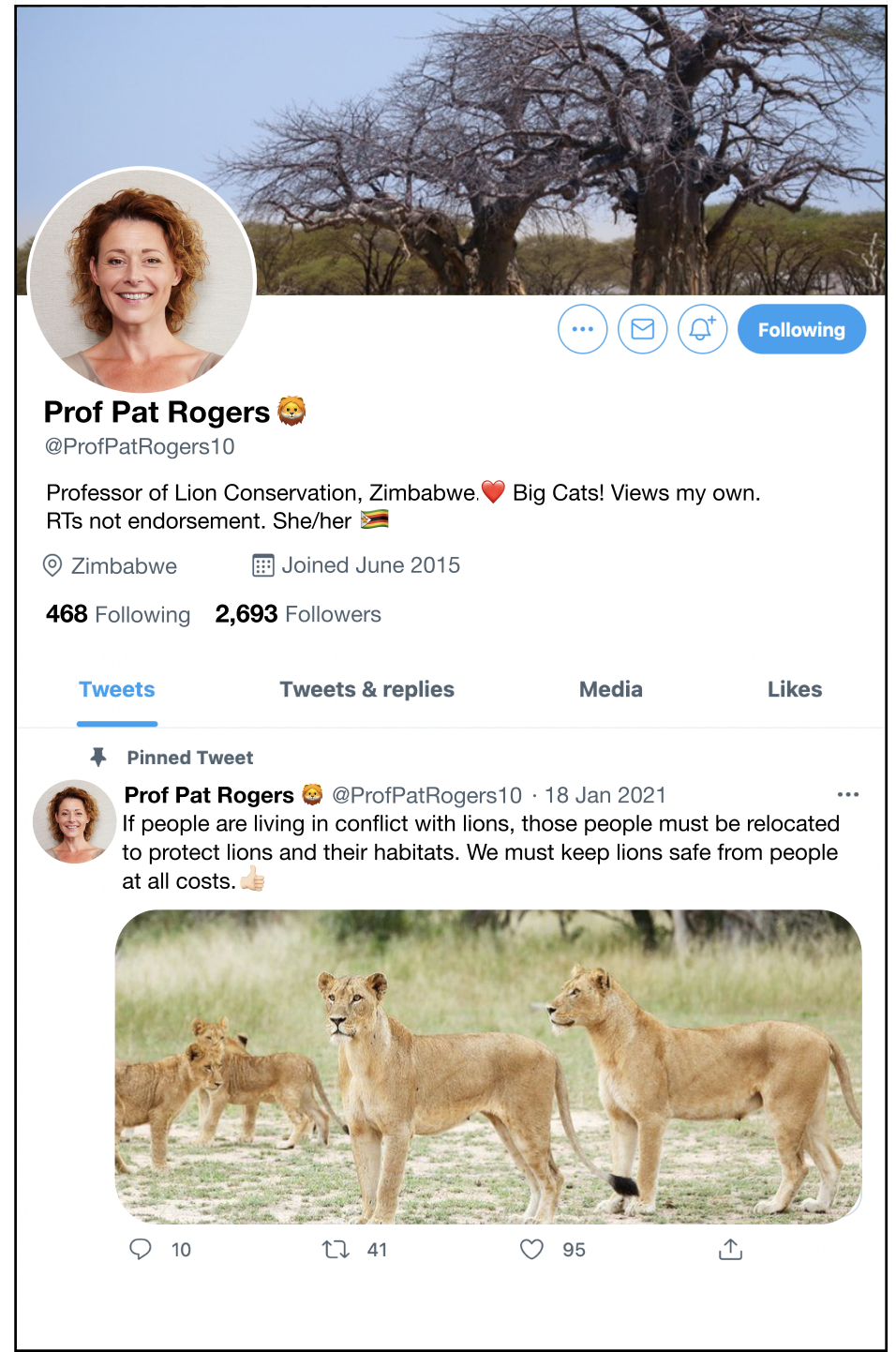


On this page you will see the Twitter profile of Pat Rodgers, who works in lion conservation, and you will be asked questions about the information their profile contains.

Your responses will help us understand how people respond to information about lion conservation on Twitter.

Please indicate how strongly you agree or disagree with each statement. If you are indifferent, please select "neither agree nor disagree". If you do not know whether you agree or disagree, please select "I don't know".

| *Item number* | *Question* | *Response options* |
| --- | --- | --- |
| 3.1 | To what extent do you agree that the information presented in Pat Rodgers’ pinned tweet is credible? | · Strongly disagree  · Disagree  · Somewhat disagree  · Neither agree nor disagree  · Somewhat agree  · Agree  · Strongly agree  · I don’t know |
| 3.2 | To what extent do you agree with Pat Rodgers that we should relocate people to address human-lion conflict? | · Strongly disagree  · Disagree  · Somewhat disagree  · Neither agree nor disagree  · Somewhat agree  · Agree  · Strongly agree  · I don’t know |
| 3.3 | To what extent do you agree that Pat Rodgers is a trustworthy source of lion conservation information? | · Strongly disagree  · Disagree  · Somewhat disagree  · Neither agree nor disagree  · Somewhat agree  · Agree  · Strongly agree  · I don’t know |

**SECTION 4: DEMOGRAPHICS AND SOCIAL IDENTITY**

| On this page you will answer some general questions about your background and identity. You answered some of these questions before entering the study, and we are asking you to answer them again now as part of the study.    By answering these questions as accurately as you can, you will help us understand how people from different backgrounds and identities interact with information about lion conservation on Twitter. All information you provide is confidential. | | | |
| --- | --- | --- | --- |
| *Item number* | *Measuring* | *Item* | *Response options* |
| 4.1 | Age | What is your age in years? | · Whole numbers between 18 and 110 only |
| 4.2 | Formal education | What is the highest level of formal education you have completed? | · Primary school  · Secondary School  · College or university degree  · Postgraduate degree |
| 4.3 | Social identity: social justice | I think of myself as someone who supports equal access, rights, and opportunities for everyone. Do you… | · Strongly disagree  · Disagree  · Somewhat disagree  · Neither agree nor disagree  · Somewhat agree  · Agree  · Strongly agree  · I don’t know |
| 4.4 | Social identity: human rights | I think of myself as someone who supports human rights. Do you… | · Strongly disagree  · Disagree  · Somewhat disagree  · Neither agree nor disagree  · Somewhat agree  · Agree  · Strongly agree  · I don’t know |
| 4.5 | Social identity: animal rights | I think of myself as someone who supports animal rights. Do you… | · Strongly disagree  · Disagree  · Somewhat disagree  · Neither agree nor disagree  · Somewhat agree  · Agree  · Strongly agree  · I don’t know |
| 4.6 | People versus animals orientation | If the interests of wild animals clash with the interests of people, which do you think should be prioritized? | · Strongly prioritize wild animals  · Prioritize wild animals  · Prioritize neither wild animals nor people  · Prioritize people  · Strongly prioritize people  · I don’t know |
| 4.7 | Trust in expertise | How much confidence do you have in each of the following to advise, make decisions and act in the best interest of the public?  a) research scientists  b) elected officials/government agency workers  c) NGO/private sector workers | · No confidence  · A little confidence  · A moderate amount confidence  · A lot of confidence  · A great deal confidence  · I don’t know |

**SECTION 5: LEAVING PAGE**

Thank you very much for participating in this study. Please click "submit" to record your responses.

This project has been reviewed by, and received ethics clearance through, the University of Oxford Central University Research Ethics Committee [reference number R79948/RE001].

The principal researcher is Lauren Rudd, who works in the Wildlife Conservation Research Unit in the Zoology Department at the University of Oxford.

If you have a concern about any aspect of this study, please email Lauren Rudd (lauren.rudd@gtc.ox.ac.uk), and we will do our best to answer your query. We will acknowledge your concern within 10 working days and give you an indication of how we will deal with it. If you remain unhappy or wish to make a formal complaint, please contact the Chair of the Medical Sciences Interdivisional Research Ethics Committee at the University of Oxford who will seek to resolve the matter as soon as possible: Email: ethics@medsci.ox.ac.uk.

**Appendix S2**

Descriptive statistics of participants in the final data set for the main study (n= 1290), broken down by demographic characteristics and social identities.

| **Characteristic** | **Responses** | **Count (%)** |
| --- | --- | --- |
|  |  |  |
| Gender Identity | Woman | 659 (51.1) |
|  | Man | 625 (48.4) |
|  | Non-binary | 5 (0.4) |
|  | Prefer not to say | 1 (0.1) |
|  |  |  |
| Age Group | 18-29 | 244 (18.9) |
|  | 30-44 | 324 (25.1) |
|  | 45-59 | 322 (25.0) |
|  | 60+ | 400 (31.0) |
|  |  |  |
| Ethnicity of respondent | White | 1110 (86.0) |
|  | *English/Welsh/Scottish/Northern Irish/British* | *730 (56.6)* |
|  | *Irish* | *69 (5.3)* |
|  | *Gypsy or Irish Traveller* | *5 (0.4)* |
|  | *Any other White background* | *306 (23.7)* |
|  | Asian/Asian British | 86 (6.6) |
|  | *Indian* | *25 (1.9)* |
|  | *Pakistani* | *4 (0.3)* |
|  | *Bangladeshi* | *0 (0)* |
|  | *Chinese* | *22 (1.7)* |
|  | *Any other Asian/Asian British background* | *35 2.7)* |
|  | Black/African/Caribbean/Black British | 44 (3.4) |
|  | *African* | *28 ( 2.2)* |
|  | *Caribbean* | *4 (0.3)* |
|  | *Any other Black/African/Caribbean background* | *12 (0.9)* |
|  | Mixed/multiple ethnic groups | 31 (2.3) |
|  | *White and Black Caribbean* | *4 (0.3)* |
|  | *White and Black African* | *7 (0.5)* |
|  | *White and Asian* | *7 (0.5)* |
|  | *Any other Mixed/Multiple ethnic groups background* | *13 (1.0)* |
|  | Other ethnic groups | 10 (0.8) |
|  | *Arab* | *1 (0.1)* |
|  | *Any other ethnic group* | *9 (0.7)* |
|  | Prefer not to say | 9 (0.7) |
|  |  |  |
| Twitter usage | Less than once a month | 89 (6.9) |
|  | At least once a month | 153 (11.9) |
|  | At least once a week | 498 (38.6) |
|  | Every day | 550 (42.6) |
|  |  |  |
| Highest level of formal education | Primary school | 39 (3.0) |
|  | Secondary school | 413 (32.0) |
|  | College or University undergraduate degree | 647 (50.2) |
|  | Postgraduate degree | 174 (13.5) |
|  | Prefer not to say | 17 ( 1.3) |
|  |  |  |
|  |  |  |
| Self reported social identity: support for human rights | Strongly disagree | 10 ( 0.8) |
|  | Disagree | 4 ( 0.3) |
|  | Somewhat disagree | 3 ( 0.2) |
|  | Neither agree nor disagree | 41 ( 3.2) |
|  | Somewhat agree | 122 ( 9.5) |
|  | Agree | 406 (31.5) |
|  | Strongly agree | 703 (54.5) |
|  | I don't know | 1 ( 0.1) |
|  |  |  |
| Self reported social identity: support for animal rights | Strongly disagree | 10 ( 0.8) |
|  | Disagree | 3 ( 0.2) |
|  | Somewhat disagree | 9 ( 0.7) |
|  | Neither agree nor disagree | 61 ( 4.7) |
|  | Somewhat agree | 153 (11.9) |
|  | Agree | 387 (30.0) |
|  | Strongly agree | 665 (51.6) |
|  | I don't know | 2 ( 0.2) |
|  |  |  |
| Prioritise people or wild animals | Strongly prioritize wild animals | 174 (13.5) |
|  | Prioritize wild animals | 332 (25.7) |
|  | Prioritize neither wild animals nor people | 260 (20.2) |
|  | Prioritize people | 276 (21.4) |
|  | Strongly prioritize people | 163 (12.6) |
|  | I don't know | 85 ( 6.6) |
|  |  |  |
| Confidence in research scientists to act in public interest | None at all | 41 ( 3.2) |
|  | A little | 122 ( 9.5) |
|  | A moderate amount | 333 (25.8) |
|  | A lot | 331 (25.7) |
|  | A great deal | 440 (34.1) |
|  | I don't know | 23 ( 1.8) |

**Appendix S3- Message selection preliminary study**

To determine which lion conservation message to use in the pinned post we first conducted a preliminary message-selection study.

## **Methods**

We tested the response to four possible messages, each of which reflected tangible strategies to address existing lion conservation problems (see Appendix S3.1). The aim was to select a message that generated responses with a close to neutral central tendency and that was not obviously polarizing (i.e. with a mean value away from either extreme and with low variance). This would allow us to best interpret any effect of bias in the main study.

All authors were involved in the process of generating these proposed messages and designing the preliminary questionnaire (Appendix S3.2)**.** Our study received ethics clearance through the University of Oxford Central University Research Ethics Committee (reference R79948/RE001).

We ran the study on the Qualtrics platform between 31 May and 7 June 2022. A total of 500 participants were recruited using Prolific. Participants were aged 18 or over and lived in the UK. All provided informed consent before starting the questionnaire and received compensation for taking part. All respondents that completed the survey in less than half the median duration time were removed from the final analysis (see Appendix S3.3).

Respondents were first presented with a short block of text about lion conservation and management strategies, to ensure they understood the topic sufficiently to answer the questionnaire, irrespective of their own prior knowledge (included with the full questionnaire in Appendix S1). Each participant was randomly presented with one of the four messages in Appendix S3.1 and was asked the extent to which they agreed with the lion conservation recommendation, using a seven-point bipolar Likert-type scale to record responses, from strongly disagree to strongly agree, with an additional “I don’t know” option. Prior to analysis, we converted responses to a numeric scale (strongly disagree = 1, strongly agree = 7) and removed “I don’t know” responses. Respondents also answered several demographic questions (gender, age, ethnicity, and level of formal education) as well as a block of questions about their social identities (extent of support for animal rights, human rights, and trust in scientists to act in the best interest of the public).

After the omission of those who completed the survey in less than half the median time, (163.5 seconds) to remove potentially unreliable responses from those who did not properly engage with the questions, the total number of respondents across the survey was n= 469. We analyzed data using the “likert” package in R (and calculated the mean and standard deviation for each message. These variables were compared across messages, and that with the fewest “I don’t know” responses, normally distributed data with low standard deviation, and with a mean response closest to 3 or 5 (somewhat agree or somewhat disagree) was chosen for use in the main study. We chose not to use a message which garnered a neutral response (mean close to 4) as this could be indicative of a topic that does not generate any opinion and so would not be influenced by identity bias.

## **Message selection results**

The acceptability of the messages about different lion conservation strategies varied considerably (Appendix S3.4). Both messages about trophy hunting generated highly skewed data with 88% of people strongly agreeing, agreeing, or somewhat agreeing that we should prevent trophy hunting (M = 5.983, SD = 1.119), and 83% of people strongly disagreeing, disagreeing, or somewhat disagreeing that we should permit trophy hunting (M= 2.244, SD= 1.562) (Appendix S3.5). As such, these messages were excluded from consideration for use in the main study.

The “Relocate lions” message generated slightly skewed data with 61% of people strongly agreeing, agreeing, or somewhat agreeing, while 28% strongly disagreed, disagreed, or somewhat disagreed (M= 4.593, SD= 1.492) (Appendix S3.5).

The “Relocate people” message generated normally distributed data with 64% strongly agreeing, agreeing, or somewhat agreeing, while 19% strongly disagreed, disagreed, or somewhat disagreed (M = 4.917, SD = 1.498) (Appendix S3.5).

We therefore selected the “Relocate people” message for use in the main study.

**Appendix S3.1**

The four candidate lion conservation messages that were tested, with participants randomly assigned one message and asked the extent to which they agreed with the statement using a 7-point Likert scale.

| Message 1 | We need to protect lions and keep them safe from people. Preventing trophy hunting will help achieve this, even if some people lose their livelihoods. |
| --- | --- |
| Message 2 | We need to protect people and their livelihoods and keep them safe from lions. Permitting trophy hunting will help achieve this, even if some lions lose their lives. |
| Message 3 | If people are living in conflict with lions, those people must be relocated to protect lions and their habitats. We must keep lions safe from people at all costs. |
| Message 4 | If people are living in conflict with lions, those lions must be relocated to protect people’s safety and livelihoods. We must keep people safe from lions at all costs. |

**Appendix S3.2- Full message selection study questionnaire**

**SECTION 1: LANDING PAGE**

Please read through the information below about our study before deciding whether to participate. You may ask any questions before deciding to take part by contacting the principal researcher (details below).

By participating in this online survey about lion conservation recommendations (it will take approximately 5-10 minutes), you will help us understand how people respond to information about lion conservation. You do not need any specialist background knowledge to participate. Our results from this study might help to inform how conservationists frame messages about lion conservation.

We will store the responses you provide in a password-protected electronic file. We will not ask you to provide any information that would identify you and we will not store your IP address. Only researchers working directly on this study will have access to the information you provide.

The data you provide may be transferred to, stored and/or processed at a destination outside the UK and the European Economic Area ("EEA"). By submitting data, you agree to this transfer, storing or processing. We may use the information you provide in academic publications such as reports and journal articles, but we will only analyze and report responses in general terms.

Qualtrics LLC is the data controller with respect to your personal data and, as such, will determine how your personal data is used. Please see their privacy notice here [www.qualtrics.com/privacy-statement/]. Qualtrics LLC will share only de-identified data with the University of Oxford, for the purposes of research.

If you choose to participate, we will first ask some questions to determine whether you are eligible for the study.

You can choose to withdraw for any reason at any point during the study by closing your browser tab. You will receive payment for participation if you complete the study, which includes answering some required questions. Some parts of the questionnaire mention people and/or animals dying, but there are no pictures or graphic descriptions.

This project has been reviewed by, and received ethics clearance through, a subcommittee of the University of Oxford Central University Research Ethics Committee [reference number R79948/RE001].

The principal researcher is Lauren Rudd, who works in the Wildlife Conservation Research Unit in the Zoology Department at the University of Oxford. This study is being conducted in collaboration with other researchers at the University of Oxford and Cornell University.

If you have a concern about any aspect of this study, please email Lauren Rudd (lauren.rudd@gtc.ox.ac.uk) and we will do our best to answer your query. We will acknowledge your concern within 10 working days and give you an indication of how we will deal with it. If you remain unhappy or wish to make a formal complaint, please contact the Chair of the Medical Sciences Interdivisional Research Ethics Committee at the University of Oxford who will seek to resolve the matter as soon as possible: Email: ethics@medsci.ox.ac.uk; Address: Research Services, University of Oxford, Boundary Brook House, Churchill Drive, OX3 7GB.

By confirming you are 18 or older and selecting "Yes, I agree to take part" below, you indicate that you voluntarily agree to participate in this study.

**Please note that you may only participate in this study only if you are 18 years of age or over.**

☐ I certify that I am 18 years of age or over

**If you have read the information above and agree to participate with the understanding that the data you submit will be processed accordingly, please check the relevant box below to get started.**

☐ Yes, I agree to take part

**SECTION 2: BACKGROUND/CONTEXT PAGE**

Please carefully read the information below, which describes the current situation surrounding lion conservation and management.

It is estimated that there are as few as 23,000 wild lions living in Africa today, around half as many as there were 20 years ago. Many lions live in protected areas such as national parks. As lions require large areas, they often move into land surrounding protected areas, where people live.

More than a quarter of the area where lions still exist is entirely outside of protected areas, alongside people. Many of the people that live alongside lions keep livestock such as goats and cows, which are essential to their livelihoods.

Some of the biggest threats to lions today are the loss of wild habitat and prey, and human-lion conflict, for example, when people legally or illegally kill lions to protect themselves or their livestock.

Moving “problem lions” that prey on livestock to a different place can sometimes reduce human-lion conflict in the original area. However, doing so can also increase human-lion conflict in the area where the lions are relocated, with risks for both people (who might suffer attacks) and lions (who might then be killed).

Lion conservation is expensive, and in most places, photo-tourism alone cannot cover the costs of effective conservation. Legal, regulated trophy hunting of lions can generate additional funding to incentivize conservation and may create local income and jobs.

Some people believe that trophy hunting lions is acceptable. However, others believe that killing lions, especially for recreation or trophies, is unacceptable.

When you have read this information, please click “Next”.

**SECTION 3: MESSAGE SELECTION**

*Each respondent was presented with the instructions below, and only one of the “item numbers” from 3.1 to 3.4.*

On this page you will read a short statement about lion conservation.

Please indicate the extent to which you agree with the conservation recommendation highlighted in the statement. If you are indifferent, please select "neither support nor oppose". If you do not know whether you support it or oppose it, please select "I don't know".

| *Item number* | *Message* | *Response options* |
| --- | --- | --- |
| 3.1 | We need to protect lions and keep them safe from people. Preventing trophy hunting will help achieve this, even if some people lose their livelihoods. | · Strongly disagree  · Disagree  · Somewhat disagree  · Neither agree nor disagree  · Somewhat agree  · Agree  · Strongly agree  · I don’t know |
| 3.2 | We need to protect people and their livelihoods and keep them safe from lions. Permitting trophy hunting will help achieve this, even if some lions lose their lives. | · Strongly disagree  · Disagree  · Somewhat disagree  · Neither agree nor disagree  · Somewhat agree  · Agree  · Strongly agree  · I don’t know |
| 3.3 | If people are living in conflict with lions, those people must be relocated to protect lions and their habitats. We must keep lions safe from people at all costs. | · Strongly disagree  · Disagree  · Somewhat disagree  · Neither agree nor disagree  · Somewhat agree  · Agree  · Strongly agree  · I don’t know |
| 3.4 | If people are living in conflict with lions, those lions must be relocated to protect people’s safety and livelihoods. We must keep people safe from lions at all costs. | · Strongly disagree  · Disagree  · Somewhat disagree  · Neither agree nor disagree  · Somewhat agree  · Agree  · Strongly agree  · I don’t know |

**SECTION 4: DEMOGRAPHICS AND SOCIAL IDENTITY**

On this page you will answer some general questions about your background and identity.

By answering these questions as accurately as you can, you will help us understand how people from different backgrounds and identities interact with information about lion conservation. All information you provide is confidential.

| *Item number* | *Measuring* | *Item* | *Response options* |
| --- | --- | --- | --- |
| 4.1 | Gender | Which best describes how you identify your gender? | · Woman  · Man  · Non-binary  · In another way  · Prefer not to say  · |
| 4.2 | Age | How old are you? | · 18-29  · 30-44  · 45-59  · 60 or older  · Prefer not to say  · |
| 4.3 | Ethnicity | Which best describes how you identify your ethnicity? | White  · English/Welsh/Scottish/Northern Irish/British  · Irish  · Gypsy or Irish Traveler  · Any other White background  Asian/Asian British  · Indian  · Pakistani  · Bangladeshi  · Chinese  · Any other Asian background  Black/African/Caribbean/Black British  · African  · Caribbean  · Any other Black/African/Caribbean background  Mixed/Multiple ethnic groups  · White and Black Caribbean  · White and Black African  · White and Asian  · Any other Mixed/Multiple ethnic background  Other ethnic group  · Arab  · Any other ethnic group    · Prefer not to say |
| 4.4 | Formal education | What is the highest level of formal education you have completed? | · Primary school  · Secondary School  · College or university degree  · Postgraduate degree |
| 4.5 | Social identity: social justice | I think of myself as someone who supports equal access, rights, and opportunities for everyone. Do you… | · Strongly disagree  · Disagree  · Somewhat disagree  · Neither agree nor disagree  · Somewhat agree  · Agree  · Strongly agree  · I don’t know |
| 4.6 | Social identity: human rights | I think of myself as someone who supports human rights. Do you… | · Strongly disagree  · Disagree  · Somewhat disagree  · Neither agree nor disagree  · Somewhat agree  · Agree  · Strongly agree  · I don’t know |
| 4.7 | Social identity: animal rights | I think of myself as someone who supports animal rights. Do you… | · Strongly disagree  · Disagree  · Somewhat disagree  · Neither agree nor disagree  · Somewhat agree  · Agree  · Strongly agree  · I don’t know |
| 4.8 | Trust in expertise | How much confidence do you have in each of the following to advise, make decisions and act in the best interest of the public?  a) research scientists  b) elected officials/government agency workers  c) NGO/private sector workers | · No confidence  · Very little confidence  · Some confidence  · A lot of confidence  · Full confidence  · I don’t know |

**SECTION 5: LEAVING PAGE**

Thank you very much for participating in this study. Please click "submit" to record your responses.

This project has been reviewed by, and received ethics clearance through, the University of Oxford Central University Research Ethics Committee [reference number R79948/RE00].

The principal researcher is Lauren Rudd, who works in the Wildlife Conservation Research Unit in the Zoology Department at the University of Oxford.

If you have a concern about any aspect of this study, please email Lauren Rudd (lauren.rudd@gtc.ox.ac.uk), and we will do our best to answer your query. We will acknowledge your concern within 10 working days and give you an indication of how we will deal with it. If you remain unhappy or wish to make a formal complaint, please contact the Chair of the Medical Sciences Interdivisional Research Ethics Committee at the University of Oxford who will seek to resolve the matter as soon as possible: Email: ethics@medsci.ox.ac.uk.

**Appendix S3.3**

Descriptive statistics of participants in the preliminary study data set (n= 469), broken down by demographic characteristics and social identities.

| **Characteristic** | **Responses** | **Count (%)** |
| --- | --- | --- |
| Gender Identity | Woman | 227 (48.4) |
|  | Man | 238 (50.7) |
|  | Non-binary | 3 (0.6) |
|  | In another way | 1 (0.2) |
|  |  |  |
| Ethnicity (%) | White | 418 (89.1) |
|  | *English/Welsh/Scottish/Northern Irish/British* | *372 (79.3)* |
|  | *Irish* | *2 (0.4)* |
|  | *Any other White background* | *44 (9.4)* |
|  | Asian/Asian British | 23 (4.9) |
|  | *Indian* | *6 (1.3)* |
|  | *Pakistani* | *3 (0.6)* |
|  | *Bangladeshi* | *1 (0.2)* |
|  | *Chinese* | *5 (1.1)* |
|  | *Any other Asian/Asian British background* | *8 (1.7)* |
|  | Black/African/Caribbean/Black British | 12 (2.6) |
|  | *African* | *8 (1.7)* |
|  | *Caribbean* | *4 (0.9)* |
|  | Mixed/multiple ethnic groups | 15 (3.3) |
|  | *White and Black Caribbean* | *4 (0.9)* |
|  | *White and Black African* | *4 (0.9)* |
|  | *White and Asian* | *4 (0.9)* |
|  | *Any other Mixed/Multiple ethnic groups background* | *3 (0.6)* |
|  | Prefer not to say | 2 (0.4) |
|  |  |  |
| Age Group | 18-29 | 136 (29.0) |
|  | 30-44 | 201 (42.9) |
|  | 45-59 | 91 (19.4) |
|  | 60+ | 41 (8.7) |
|  |  |  |
| Highest level of formal education | Primary school | 2 (0.4) |
|  | Secondary school | 104 (22.2) |
|  | College or University undergraduate degree | 255 (54.4) |
|  | Postgraduate degree | 106 (22.6) |
|  | Prefer not to say | 2 (0.4) |
|  |  |  |
| Self-reported social identity: support for human rights | Strongly disagree | 2 (0.4) |
|  | Neither agree nor disagree | 12 (2.6) |
|  | Somewhat agree | 44 (9.4) |
|  | Agree | 171 (36.5) |
|  | Strongly agree | 240 (51.2) |
|  |  |  |
| Self-reported social identity: support for animal rights | Strongly disagree | 1 (0.2) |
|  | Disagree | 3 (0.6) |
|  | Somewhat disagree | 5 (1.1) |
|  | Neither agree nor disagree | 22 (4.7) |
|  | Somewhat agree | 70 (14.9) |
|  | Agree | 166 (35.4) |
|  | Strongly agree | 202 (43.1) |
|  |  |  |
| Confidence in research scientists to act in public interest | None at all | 11 ( 2.3) |
|  | A little | 23 ( 4.9) |
|  | A moderate amount | 98 (20.3) |
|  | A lot | 131 (27.9) |
|  | A great deal | 202 (43.1) |
|  | I don't know | 7 (1.5) |

**Appendix S3.4**

**Agreement with each of the tested lion conservation messages.** Likert plot showing responses to all four candidate messages. Bars represent one message each and show the distribution of responses, coloured from reds (disagreement), through grays (neutrality), to blues (agreement). The percentage figures show the number of participants who overall disagreed (left), were neutral (middle), or agreed (right) after removing “I don’t know” responses.


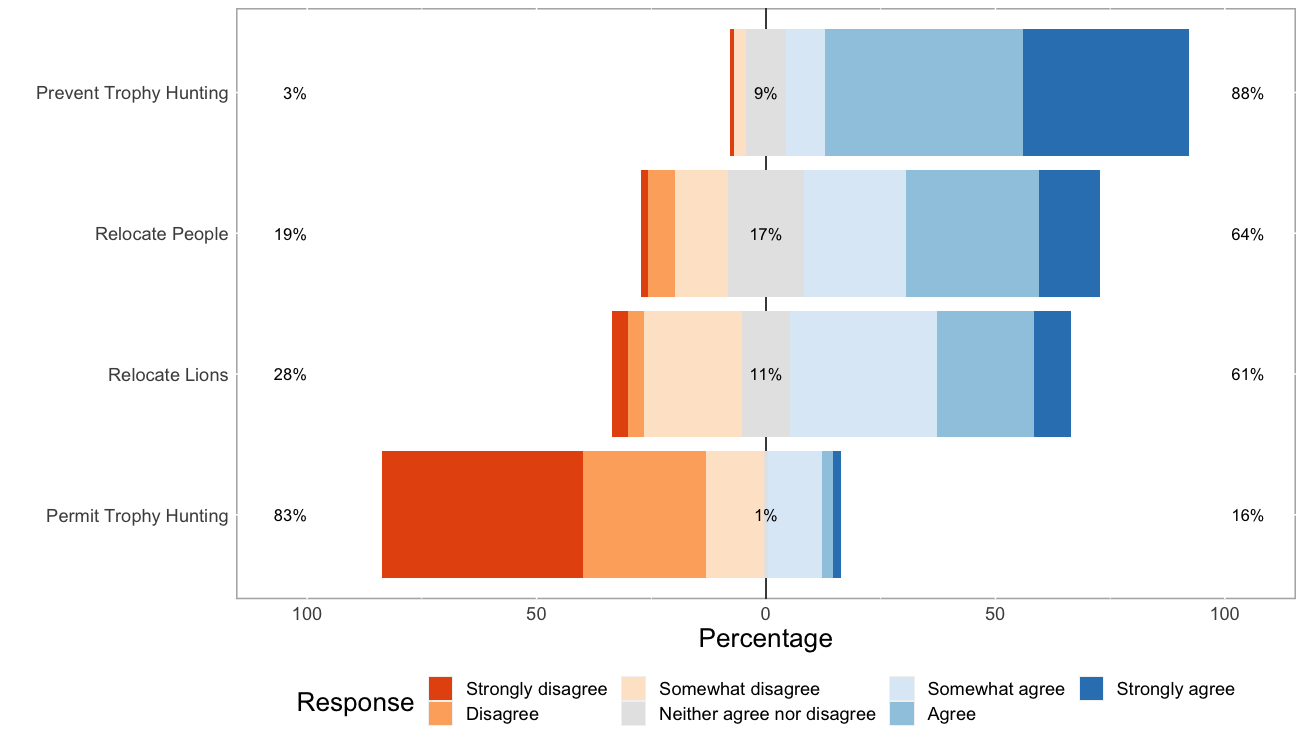


**Appendix S3.5**

Results of the message selection study showing the mean response (ranging from 1= strongly disagree to 7= strongly agree), standard deviation, and number of respondents for each of the four options tested.

|  | **Mean** | **SD** | **N** |
| --- | --- | --- | --- |
| Prevent Trophy Hunting | 5.983 | 1.119 | 116 |
| Permit Trophy Hunting | 2.244 | 1.562 | 119 |
| Relocate People | 4.917 | 1.498 | 121 |
| Relocate Lions | 4.593 | 1.492 | 113 |

**Appendix S4.1- AIC weights table for question 1**

Results of model selection process for question 1 after removing redundant and uninformative parameters, showing the top-supported model and all models within Δ2 AIC_c_.

| **Model** | **df** | **logLik** | **AIC_c_** | **delta** | **AIC_w_** |
| --- | --- | --- | --- | --- | --- |
| age + animal rights + respondent ethnicity + character gender + human rights + people vs animals + trust in scientists + twitter usage | 38 | -2032.706 | 4143.854 | 0 | 0.217 |
| age + animal rights + respondent ethnicity + human rights + people vs animals + trust in scientists + twitter usage | 37 | -2033.867 | 4144.047 | 0.193 | 0.197 |
| age + animal rights + respondent ethnicity + character gender + people vs animals + trust in scientists + twitter usage | 32 | -2039.228 | 4144.186 | 0.332 | 0.184 |
| age + animal rights + respondent ethnicity + people vs animals + trust in scientists + twitter usage | 31 | -2040.474 | 4144.573 | 0.719 | 0.151 |
| age + animal rights + respondent ethnicity + human rights + people vs animals + trust in scientists + twitter usage + character nationality | 38 | -2033.647 | 4145.735 | 1.881 | 0.085 |
| age + animal rights + respondent ethnicity + human rights + people vs animals + trust in scientists + twitter usage + character expertise | 39 | -2032.590 | 4145.751 | 1.897 | 0.084 |
| age + animal rights + respondent ethnicity + character gender + people vs animals + trust in scientists + twitter usage + character nationality | 33 | -2038.979 | 4145.799 | 1.945 | 0.082 |

**Appendix S4.2- AIC weights table for question 2**

Results of model selection process for question 2 after removing redundant and uninformative parameters, showing the top-supported model and all models within Δ2 AIC_c_.

| **Model** | **df** | **logLik** | **AIC_c_** | **delta** | **AIC_w_** |
| --- | --- | --- | --- | --- | --- |
| Age + animal rights + respondent ethnicity + character expertise + human rights + people vs animals + character race + trust in scientists + twitter usage + character expertise * race | 42 | -2097.886 | 4282.742 | 0 | 0.350 |
| Age + animal rights + respondent ethnicity + character expertise + people vs animals + character race + trust in scientists + twitter usage + character expertise * race | 36 | -2104.528 | 4283.236 | 0.493 | 0.273 |
| Age + animal rights + respondent ethnicity + character expertise + people vs animals + character race + trust in scientists + twitter usage + character expertise * race + character gender | 37 | -2103.572 | 4283.446 | 0.704 | 0.246 |
| Age + animal rights + character expertise + human rights + people vs animals + character race + trust in scientists + twitter usage + character expertise * race | 37 | -2104.206 | 4284.715 | 1.972 | 0.131 |

**Appendix S4.3- AIC weights table for question 3**

Results of model selection process for question 3 after removing redundant and uninformative parameters, showing the top-supported model and all models within Δ2 AIC_c_.

| **Model** | **df** | **logLik** | **AIC_c_** | **delta** | **AIC_w_** |
| --- | --- | --- | --- | --- | --- |
| Animal rights + respondent ethnicity + character expertise + character gender + people vs animals + character race + trust in scientists + twitter usage + character expertise * race + character gender * race | 37 | -1904.137 | 3884.744 | 0 | 0.234 |
| Animal rights + character expertise + character gender + people vs animals + character race + trust in scientists + twitter usage + character expertise * race + character gender * race + character nationality + character nationality * race | 34 | -1907.924 | 3885.935 | 1.191 | 0.129 |
| Animal rights + character expertise + character gender + people vs animals + character race + trust in scientists + twitter usage + character expertise * race + character gender * race | 32 | -1910.065 | 3885.979 | 1.235 | 0.126 |
| Animal rights + respondent ethnicity + character gender + people vs animals + character race + trust in scientists + twitter usage + character gender * race | 33 | -1909.032 | 3886.030 | 1.286 | 0.123 |
| Animal rights + respondent ethnicity + character gender + people vs animals + character race + trust in scientists + twitter usage + character gender * race + character nationality + character nationality * race | 35 | -1906.964 | 3886.139 | 1.395 | 0.116 |
| Animal rights + respondent ethnicity + character gender + people vs animals + character race + trust in scientists + twitter usage + character gender * race + character nationality + character nationality * race | 34 | -1908.178 | 3886.443 | 1.699 | 0.100 |
| Animal rights + respondent ethnicity + people vs animals + trust in scientists + twitter usage | 30 | -1912.515 | 3886.655 | 1.911 | 0.090 |
| Animal rights + respondent ethnicity + character expertise + character gender + people vs animals + character race + trust in scientists + twitter usage + character gender * race | 35 | -1907.300 | 3886.810 | 2.066 | 0.083 |

**Appendix S5**

Summary of the ordinal regression results from the top-supported model for question 1 (to what extent do you agree that the information presented in Pat Rogers’ pinned tweet is credible?) with coefficient estimates presented alongside the standard error, 85% confidence intervals, and p values. The baseline of reference for each categorical variable is as follows- Gender, Woman; People vs animals, Strongly prioritize animals; Animal rights, Strongly disagree; Human rights, Strongly disagree; Scientists, none at all; Twitter usage, Less than once a month; Ethnicity of respondent, White.

| **Variable** | **Coefficient estimate** | **SE** | **CI (85%)** | **p** |
| --- | --- | --- | --- | --- |
| Gender, Man | 0.157 | 0.103 | 0.009, 0.305 | 0.128 |
| Animal rights, Disagree | 1.766 | 1.499 | -0.439, 3.917 | 0.239 |
| Animal rights, Somewhat disagree | 2.953 | 1.128 | 1.331, 4.599 | 0.009 |
| Animal rights, Neither agree nor disagree | 2.024 | 0.945 | 0.671, 3.423 | 0.032 |
| Animal rights, Somewhat agree | 2.416 | 0.931 | 1.085, 3.796 | 0.009 |
| Animal rights, Agree | 2.676 | 0.927 | 1.350, 4.050 | 0.004 |
| Animal rights, Strongly agree | 3.636 | 0.925 | 2.312, 5.005 | <0.001 |
| Human rights, Disagree | -0.521 | 1.514 | -2.814, 1.550 | 0.731 |
| Human rights, Somewhat disagree | 0.248 | 1.232 | -1.578, 2.016 | 0.840 |
| Human rights, Neither agree nor disagree | 1.455 | 0.825 | 0.275, 2.657 | 0.078 |
| Human rights, Somewhat agree | 1.835 | 0.789 | 0.707, 2.986 | 0.020 |
| Human rights, Agree | 1.696 | 0.779 | 0.582, 2.835 | 0.030 |
| Human rights, Strongly agree | 1.492 | 0.774 | 0.386, 2.625 | 0.054 |
| People vs animals, Prioritize animals | -0.815 | 0.182 | -1.078, -0.555 | <0.001 |
| People vs animals, Prioritize neither animals nor people | -1.360 | 0.196 | -1.643, -1.079 | <0.001 |
| People vs animals, Prioritize people | -1.459 | 0.201 | -1.748, -1.171 | <0.001 |
| People vs animals, Strongly prioritize people | -1.207 | 0.224 | -1.530, -0.885 | <0.001 |
| People vs animals, I don't know | -1.556 | 0.257 | -1.927, -1.186 | <0.001 |
| Scientists, A little | 0.577 | 0.360 | 0.060, 1.099 | 0.109 |
| Scientists, A moderate amount | 0.521 | 0.336 | 0.039, 1.009 | 0.121 |
| Scientists, A lot | 0.964 | 0.338 | 0.479, 1.453 | 0.004 |
| Scientists, A great deal | 1.255 | 0.338 | 0.770, 1.746 | <0.001 |
| Scientists, I don't know | 0.734 | 0.523 | -0.018, 1.489 | 0.160 |
| Twitter usage, At least once a month | -0.075 | 0.245 | -0.428, 0.278 | 0.760 |
| Twitter usage, At least once a week | -0.055 | 0.212 | -0.360, 0.250 | 0.797 |
| Twitter usage, Every day | 0.454 | 0.212 | 0.148, 0.760 | 0.033 |
| Age | -0.010 | 0.003 | -0.015, -0.005 | 0.002 |
| Ethnicity of respondent, Asian | 0.472 | 0.213 | 0.166, 0.779 | 0.027 |
| Ethnicity of respondent, Black | 0.750 | 0.281 | 0.347, 1.159 | 0.008 |
| Ethnicity of respondent, Mixed race | -0.226 | 0.339 | -0.715, 0.263 | 0.504 |
| Ethnicity of respondent, Any other ethnic group | -0.415 | 0.550 | -1.209, 0.387 | 0.451 |
| Ethnicity of respondent, Prefer not to say | -0.028 | 0.604 | -0.902, 0.850 | 0.962 |

**Appendix S6.1**

Summary of the ordinal regression results from the top-supported model for question 2 (to what extent do you agree with Pat Rogers that we should relocate people to protect lions?) with coefficient estimates presented alongside the standard error, 85% confidence intervals, and p values. The baseline of reference for each categorical variable is as follows- Race, Black; Expertise, Enthusiast; Animal rights, Strongly agree; Human rights, Strongly agree; People vs animals, Strongly prioritize animals; Scientists, None at all; Twitter usage, Less than once a month; Ethnicity of respondent, White.

| **Variable** | **Coefficient estimate** | **SE** | **CI (85%)** | | **p** |
| --- | --- | --- | --- | --- | --- |
| Race, White | -0.300 | 0.179 | | -0.559, -0.042 | 0.094 |
| Expertise, Field assistant | -0.214 | 0.174 | | -0.465, 0.036 | 0.219 |
| Expertise, Professor | -0.437 | 0.177 | | -0.692, -0.183 | 0.013 |
| Race, White*Expertise, Field assistant | 0.024 | 0.251 | | -0.338, 0.386 | 0.924 |
| Race, White*Expertise, Professor | 0.727 | 0.251 | | 0.367, 1.088 | 0.004 |
| Animal rights, Disagree | -1.395 | 1.421 | | -3.677, 0.572 | 0.326 |
| Animal rights, somewhat Disagree | 0.641 | 0.969 | | -0.738, 2.071 | 0.508 |
| Animal rights, Neither agree nor disagree | 0.762 | 0.826 | | -0.403, 2.004 | 0.356 |
| Animal rights, Somewhat agree | 1.563 | 0.81 | | 0.425, 2.784 | 0.053 |
| Animal rights, Agree | 1.761 | 0.807 | | 0.627, 2.978 | 0.029 |
| Animal rights, Strongly agree | 2.680 | 0.807 | | 1.547, 3.896 | 0.001 |
| Human rights, Disagree | 0.216 | 1.104 | | -1.388, 1.810 | 0.845 |
| Human rights, Somewhat disagree | -1.263 | 1.380 | | -3.506, 0.625 | 0.360 |
| Human rights, Neither agree nor disagree | 1.009 | 0.767 | | -0.089, 2.131 | 0.188 |
| Human rights, Somewhat agree | 1.329 | 0.720 | | 0.299, 2.386 | 0.065 |
| Human rights, Agree | 1.152 | 0.713 | | 0.133, 2.199 | 0.106 |
| Human rights, Strongly agree | 0.928 | 0.708 | | -0.084, 1.970 | 0.190 |
| People vs animals, Prioritize animals | -0.996 | 0.181 | | -1.258, -0.736 | <0.001 |
| People vs animals, Prioritize neither animals nor people | -1.727 | 0.197 | | -2.011, -1.445 | <0.001 |
| People vs animals, Prioritize people | -2.051 | 0.201 | | -2.342, -1.763 | <0.001 |
| People vs animals, Strongly prioritize people | -1.841 | 0.230 | | -2.173, -1.512 | <0.001 |
| People vs animals, I don't know | -2.071 | 0.263 | | -2.451, -1.694 | <0.001 |
| Scientists, A little | 0.257 | 0.377 | | -0.283, 0.805 | 0.497 |
| Scientists, A moderate amount | 0.294 | 0.354 | | -0.212, 0.810 | 0.407 |
| Scientists, A lot | 0.729 | 0.355 | | 0.223, 1.246 | 0.040 |
| Scientists, A great deal | 0.928 | 0.355 | | 0.421, 1.444 | 0.009 |
| Scientists, I don't know | 0.953 | 0.525 | | 0.199, 1.714 | 0.070 |
| Twitter usage, At least once a month | -0.127 | 0.248 | | -0.484, 0.229 | 0.608 |
| Twitter usage, At least once a week | 0.068 | 0.218 | | -0.246, 0.382 | 0.755 |
| Twitter usage, Every day | 0.315 | 0.218 | | 0.001, 0.628 | 0.148 |
| Age | -0.018 | 0.003 | | -0.023, -0.014 | <0.001 |
| Ethnicity of respondent Asian | 0.534 | 0.209 | | 0.234, 0.837 | 0.011 |
| Ethnicity of respondent Black | 0.500 | 0.287 | | 0.086, 0.914 | 0.082 |
| Ethnicity of respondent Mixed race | 0.252 | 0.340 | | -0.238, 0.743 | 0.459 |
| Ethnicity of respondent Any other ethnic group | -0.861 | 0.519 | | -1.611, -0.106 | 0.097 |
| Ethnicity of respondent Prefer not to say | -0.226 | 0.586 | | -1.086, 0.616 | 0.700 |

**Appendix S6.2**

Summary of the Tukey test results for the interaction terms in the top-supported model for question 2 (to what extent do you agree with Pat Rogers that we should relocate people to protect lions?) with coefficient estimates presented alongside the standard error.

| **Race** | **Expertise** | **Estimate** | **SE** | **df** | **Asymp.LCL** | **Asymp.UCL** |
| --- | --- | --- | --- | --- | --- | --- |
| Black | Enthusiast | 1.076 | 0.128 | Inf | 0.689 | 1.464 |
| White | Enthusiast | 0.776 | 0.132 | Inf | 0.376 | 1.176 |
| Black | Field assistant | 0.862 | 0.125 | Inf | 0.483 | 1.241 |
| White | Field assistant | 0.586 | 0.127 | Inf | 0.200 | 0.971 |
| Black | Professor | 0.639 | 0.127 | Inf | 0.255 | 1.024 |
| White | Professor | 1.066 | 0.126 | Inf | 0.684 | 1.448 |

**Appendix S7.1**

Summary of the ordinal regression results from the top-supported model for question 3 (to what extent do you agree that Pat Rogers is a trustworthy source of lion conservation information?) with coefficient estimates presented alongside the standard error, 85% confidence intervals, and p values. The baseline of reference for each categorical variable is as follows- Race, Black; Gender, Woman; Expertise, Enthusiast; Animal rights, Strongly disagree; People vs animals, Strongly prioritize animals; Scientists, none at all; Twitter usage, Less than once a month; Ethnicity of respondent, White.

| **Variable** | **Coefficient estimate** | **SE** | **CI (85%)** | **p** |
| --- | --- | --- | --- | --- |
| Race, White | -0.012 | 0.216 | -0.322, 0.298 | 0.955 |
| Gender, Man | 0.320 | 0.149 | 0.106, 0.535 | 0.031 |
| Expertise, Field assistant | -0.112 | 0.181 | -0.372, 0.148 | 0.537 |
| Expertise, Professor | -0.116 | 0.185 | -0.383, 0.151 | 0.532 |
| Race, White*Expertise, Field assistant | 0.213 | 0.260 | -0.161, 0.587 | 0.413 |
| Race, White*Expertise, Professor | 0.646 | 0.262 | 0.268, 1.024 | 0.014 |
| Race, White*Gender, Man | -0.575 | 0.213 | -0.883, -0.268 | 0.007 |
| Animal rights, Disagree | 2.017 | 1.268 | 0.171, 3.840 | 0.112 |
| Animal rights, somewhat Disagree | 1.029 | 0.918 | -0.288, 2.365 | 0.262 |
| Animal rights, Neither agree nor disagree | 0.949 | 0.746 | -0.113, 2.047 | 0.204 |
| Animal rights, Somewhat agree | 1.891 | 0.723 | 0.864, 2.958 | 0.009 |
| Animal rights, Agree | 1.981 | 0.712 | 0.971, 3.033 | 0.005 |
| Animal rights, Strongly agree | 2.527 | 0.711 | 1.518, 3.579 | <0.001 |
| People vs animals, Prioritize animals | -0.834 | 0.185 | -1.101, -0.569 | <0.001 |
| People vs animals, Prioritize neither animals nor people | -1.285 | 0.199 | -1.572, -0.999 | <0.001 |
| People vs animals, Prioritize people | -1.159 | 0.199 | -1.446, -0.873 | <0.001 |
| People vs animals, Strongly prioritize people | -0.861 | 0.220 | -1.178, -0.545 | <0.001 |
| People vs animals, I don't know | -1.103 | 0.279 | -1.506, -0.701 | <0.001 |
| Scientists, A little | 0.789 | 0.385 | 0.236, 1.346 | 0.041 |
| Scientists, A moderate amount | 0.970 | 0.36 | 0.454, 1.492 | 0.007 |
| Scientists, A lot | 1.297 | 0.363 | 0.778, 1.823 | <0.001 |
| Scientists, A great deal | 1.493 | 0.361 | 0.976, 2.016 | <0.001 |
| Scientists, I don't know | 0.635 | 0.536 | -0.136, 1.411 | 0.237 |
| Twitter usage, At least once a month | 0.336 | 0.256 | -0.033, 0.705 | 0.190 |
| Twitter usage, At least once a week | 0.405 | 0.223 | 0.083, 0.726 | 0.070 |
| Twitter usage, Every day | 0.829 | 0.224 | 0.508, 1.152 | <0.001 |
| Ethnicity of respondent Asian | 0.445 | 0.211 | 0.142, 0.749 | 0.035 |
| Ethnicity of respondent Black | 0.587 | 0.301 | 0.156, 1.024 | 0.051 |
| Ethnicity of respondent Mixed race | -0.239 | 0.369 | -0.771, 0.294 | 0.517 |
| Ethnicity of respondent Any other ethnic group | -0.612 | 0.620 | -1.526, 0.271 | 0.324 |
| Ethnicity of respondent Prefer not to say | -0.935 | 0.630 | -1.844, -0.016 | 0.138 |

**Appendix S7.2**

Summary of the Tukey test results for the interaction terms (race*expertise, and race*gender) in the top-supported model for question 3 (to what extent do you agree that Pat Rogers is a trustworthy source of lion conservation information?) with coefficient estimates presented alongside the standard error.

| **Race** | **Expertise** | **Estimate** | **SE** | **df** | **Asymp.LCL** | **Asymp.UCL** |
| --- | --- | --- | --- | --- | --- | --- |
| Black | Enthusiast | 1.276 | 0.136 | Inf | 0.861 | 1.690 |
| White | Enthusiast | 0.974 | 0.137 | Inf | 0.558 | 1.389 |
| Black | Field assistant | 1.164 | 0.132 | Inf | 0.764 | 1.564 |
| White | Field assistant | 1.075 | 0.137 | Inf | 0.658 | 1.493 |
| Black | Professor | 1.160 | 0.137 | Inf | 0.744 | 1.575 |
| White | Professor | 1.504 | 0.137 | Inf | 1.088 | 1.919 |

| **Race** | **Gender** | **Estimate** | **SE** | **df** | **Asymp.LCL** | **Asymp.UCL** |
| --- | --- | --- | --- | --- | --- | --- |
| Black | Woman | 1.038 | 0.111 | Inf | 0.727 | 1.350 |
| White | Woman | 1.315 | 0.118 | Inf | 0.984 | 1.646 |
| Black | Man | 1.359 | 0.114 | Inf | 1.039 | 1.678 |
| White | Man | 1.060 | 0.111 | Inf | 0.749 | 1.370 |

**Appendix S8**

**Change in rank position of each “Pat Rogers” character over the three research questions.** Plot shows the ranked position of the 24 versions of Pat Rogers (based on all possible combinations of the levels of expertise, race, gender, and nationality tested), from 1 (highest agreement) to 24 (lowest agreement) for each research question. Rankings were calculated based on overall percentage of responses in agreement (inclusive of somewhat agree, agree, and strongly agree), with higher percentage agreement increasing the ranking of the profile.

**
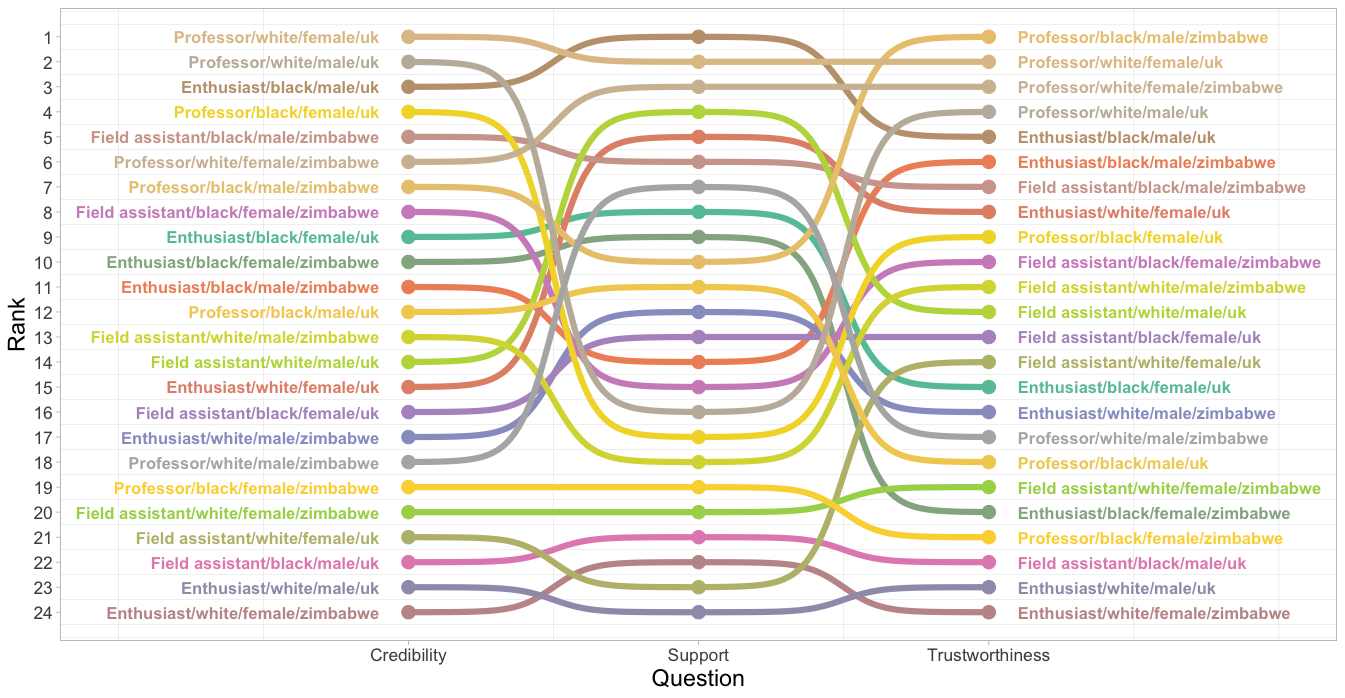
**

**Appendix S9- Extended Results and Discussion**

**Extended results**

Across all three research questions, and all 24 profiles, the greatest proportion of respondents were in overall agreement rather than disagreement, but there was considerable variation between profiles within each research question (see Figures 3a, 4a and 6a). The relative ranking of each version of Pat Rogers, based on the extent of respondent’s agreement across the three research questions, showed considerable variability (Appendix S8). The only character that consistently ranked within the top 3 was the white, woman, British professor, while the only characters to consistently rank within the bottom 3 were the white, man, British enthusiast and the white, woman, Zimbabwean enthusiast.

**Credibility**

The white, woman, British professor was viewed as the most credible source (81.1% of respondents either strongly agreeing, agreeing, or somewhat agreeing with the statement provided by this character). This character also had the highest number of participants strongly agreeing that the post was credible (32.1%). Overall, the white, woman, Zimbabwean enthusiast was perceived to be least credible (31.3% of respondents either strongly disagreeing, disagreeing, or somewhat disagreeing with their post). However, the Black, woman, Zimbabwean field assistant generated the highest proportion of respondents strongly disagreeing that the post was credible (9.8%; Figure 2a).

**Support**

The Black, man, British enthusiast generated the most support (75.4% of respondents either strongly agreeing, agreeing, or somewhat agreeing). This character also had the highest number of participants strongly agreeing that people should be relocated to address human-lion conflict (31.6%). Overall, the white, woman, Zimbabwean enthusiast, generated the least support (43.7% of respondents either strongly disagreeing, disagreeing, or somewhat disagreeing). However, the white, woman, Zimbabwean, field assistant generated the biggest proportion of respondents strongly disagreeing that people should be relocated (12.0%).

**Trustworthiness**

The Black, man, Zimbabwean professor was perceived as most trustworthy (76.4% of respondents either strongly agreeing, agreeing, or somewhat agreeing). However, the white, woman, Zimbabwean professor generated the biggest proportion of respondents strongly agreeing that Pat Rogers was trustworthy (34.7%). Overall, the Black, man, Zimbabwean enthusiast was perceived as least trustworthy (22.9% of respondents either strongly disagreeing, disagreeing, or somewhat disagreeing). However, the Black, woman, British professor generated the biggest proportion of respondents strongly disagreeing that Pat Rogers was trustworthy (9.6%).

**Extended discussion**

We chose to explore these questions using X as the platform for conservation information dissemination, primarily because it facilitated the seamless display of a conservation message (via the pinned post) beside the identity details we manipulated in the biography. This provided a unique set up that would be difficult to replicate via other social media platforms or news outlets.

We further chose to contextualize the study within the field of lion conservation due to the charismatic status of the species. Recently there has been considerable news coverage surrounding lion conservation in the UK, specifically focused on trophy hunting bans [(Yeomans et al., 2022)](https://www.zotero.org/google-docs/?8nj1A0). While we did attempt to control for discrepancies in individual knowledge of lion conservation by providing an objective statement at the start of the survey (Appendix S1), it is possible that existing opinions will have influenced participants' responses. Overall, most respondents agreed with the credibility of the lion conservation information, supported its implementation, and trusted the conservationist as a source of information. It would be interesting to conduct a similar study using a less charismatic species, or using a message that generated less public support, to investigate what effect these factors have on identity- bias.

With increasing age, both perceived credibility and support for the conservationist’s recommendation decreased. This could be due to greater skepticism towards scientific information on X amongst older demographics, or a skepticism towards trusting information communicated by individuals younger than themselves.

The ethnicity of participants influenced their response to each of our research questions. We interpret these results with caution because of the low sample sizes within several of the ethnic groups (due to stratifying the respondent pool to match the UK population). In future it would be helpful to sample equally across ethnic groups to facilitate a more detailed analysis of these effects.
